# 3D Printing of Multiscale Ti64‐Based Lattice Electrocatalysts for Robust Oxygen Evolution Reaction

**DOI:** 10.1002/advs.202201751

**Published:** 2022-07-20

**Authors:** Binbin Guo, Jiahui Kang, Tianbiao Zeng, Hongqiao Qu, Shixiang Yu, Hui Deng, Jiaming Bai

**Affiliations:** ^1^ Department of Mechanical and Energy Engineering Southern University of Science and Technology Shenzhen 518055 China; ^2^ School of Chemistry and Materials Engineering Wenzhou University Wenzhou 325035 China

**Keywords:** 3D Printing, lattice, oxygen evolution reaction, selective laser melting, Ti–6Al–4V titanium alloy

## Abstract

Electrically assisted water splitting is an endurable strategy for hydrogen production, but the sluggish kinetics of oxygen evolution reaction (OER) extremely restrict the large‐scale production of hydrogen. Developing highly efficient and non‐precious catalytic materials is essential to accelerate the sluggish kinetics of OER. However, currently used catalyst supports, such as copper foam, suffer from inferior corrosion resistance and structural stability, resulting in the disabled functionality of 3D conductive networks. To this end, a novel 3D freestanding electrode with corrosion‐resistant and robust Ti–6Al–4V titanium alloy lattice as the catalyst support is designed via a 3D printing technology of selective laser melting. After the coating of core–shell Cu(OH)2@CoNi carbonate hydroxides (CoNiCH) on the designed lattice, a unique micro/nano‐sized hierarchical porous structure is formed, which endows the electrocatalyst with a promising electrocatalytic activity (a low overpotential of 355 mV at 30 mA cm^−2^ and Tafel slope of 125.3 mV dec^−1^). Computational results indicate that the CoNiCH exhibits optimized electron transfer and the catalytic activity of the Ni site is higher than that of the Co site in the CoNiCH. Therefore, the integration of robust catalyst supports and highly active materials opens up an avenue for reliable and high‐performance OER electrocatalysts.

## Graphical abstract

A corrosion‐resistant and mechanically robust Ti–6Al–4V porous catalyst substrate is fabricated by selective laser melting 3D printing. After the coating of Cu(OH)_2_@CoNi carbonate hydroxides, a multiscale porous structure is designed for promising electrocatalytic performance including electrochemical stability and mechanical stability. Density functional theory and density of state calculations reveal the catalytically active sites and optimized electron transfer of the electrocatalysts.

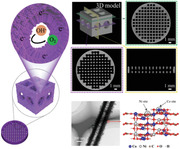

## 1 Introduction

Electrocatalytic water splitting provides a scheme to solve the challenges, in which hydrogen and oxygen (O_2_) are converted from electrical energy, serving as fuel and feedstock for chemical industries.^[^ ^1^ ^]^ However, owing to a four‐electron reaction, the anodic oxygen evolution reaction (OER) exhibits a higher energy barrier than the cathodic hydrogen evolution reaction with a two‐electron reaction, which is regarded as a rate‐determining step in electrochemical splitting water processes.^[^ ^2^ ^]^ Previous studies have reported that noble metal and their oxides, such as Pd, IrO_2_, and RuO_2_, exhibit excellent performance in an electrochemical activity.^[^ ^3^ ^]^ However, the high cost and scarce resources limit the extensive use of noble metal catalysts in water splitting. As an alternative, the transition metal (i.e., Ni, Co, Fe, and Mn) oxides, hydroxides, chalcogenides, and their compounds have aroused great scientific interest due to their low‐cost abundance and low toxicity.^[^ ^4^ ^]^ Recently, developing bimetallic carbonate hydroxide (BCH) electrocatalysts is a practical strategy for boosting the catalytic activity due to the optimized electronic structures.^[^ ^5^ ^]^ For example, CoNi carbonate hydroxides (CoNiCH) catalysts have been broadly investigated in water oxidation applications.^[^ ^6^ ^]^ In addition to the design of double transition metal elements, the rational design of morphologies and microstructures of catalysts is essential to further increase the intrinsic activities. For instance, developing tubular core–shell heterostructures with a core of Cu(OH)_2_ and shells of CoNiCH can reduce the resistance along the longitudinal direction and enlarge the surface area for contacting the electrolyte, which is beneficial to improving electrochemical activity.^[^ ^7^ ^]^

Apart from the development of highly active materials, enhancing exposed active sites is another strategy for increasing catalytic activity. Generally, to get a large active site exposure, a thick catalyst bulk is introduced to obtain a high mass loading of catalysts. However, the bubble removal and electrolyte circulation in a thick electrode are impeded, which negatively affects the catalytic activity.^[^ ^8^ ^]^ Therefore, constructing 3D porous catalyst supports for active materials is essential to expose large active sites and provide hollow structures for rapid bubble removal and electrolyte circulation.^[^ ^9^ ^]^ For instance, in situ growth of catalysts on porous copper (Cu) foam exhibits promising electrocatalytic activity.^[^ ^7^, ^10^ ^]^ However, as one of the most currently used catalyst supports, Cu foam has several limitations: i) Cu foam easily oxidizes and corrodes, which degrades the electrical conductivity; ii) the inferior mechanical stability of Cu foam will limit the long‐term use of catalysts. To endow OER catalysts with reliable electrochemical performance, it is crucial to design and fabricate 3D porous, corrosion‐resistant, and mechanically robust catalyst supports. However, 3D supports with complex structures cannot be controllably fabricated by conventional methods. 3D printing exhibits advantages in high geometry freedom and easy handleability to construct designable fine architecture as it is an emerging manufacturing technology for fabricating components in a layer‐by‐layer manner.^[^ ^11^ ^]^

As a powder‐based 3D printing technology, selective laser melting (SLM) utilizes a laser beam to selectively melt powders into predefined 3D geometries.^[^ ^12^ ^]^ The main metal powders utilized in the SLM process are Ti–6Al–4V (Ti64) powders, and the fabricated Ti64 components have been applied in implant, aerospace, and electronics applications due to their superior biocompatibility, mechanical, and electrical properties, respectively.^[^ ^12^, ^13^ ^]^ Additionally, Ti64 alloy exhibits outstanding corrosion resistance to meet the requirements of severe environments.^[^ ^14^ ^]^ Therefore, the Ti64 alloy is ideal catalyst support for electrocatalysts, in which the SLM‐printed Ti64 alloy possesses tailorable geometries to improve the electrocatalytic reactions at the gas–liquid–solid phase boundaries and promising electrical stability to guarantee the functionality of electrocatalysts. Nevertheless, up to date, there is no report about the SLM‐printed Ti64 alloy used for OER catalyst supports in the literature.

Herein, we presented an integrated OER electrocatalyst containing the SLM‐printed hollow Ti64 lattice as the catalyst support and subsequently coated core–shell Cu(OH)_2_@CoNiCH as the active materials. Distinct from previous reports, the introduction of Ti64 catalyst substrate endowed the OER catalyst with impressive corrosion resistance in alkaline media and promising mechanical stability, which guaranteed the durability of electrocatalysts. Additionally, employing the density functional theory (DFT) and density of state (DOS) calculations, the catalytically active sites and optimized electron transfer of the CoNiCH were revealed for the first time. This design philosophy of correlating 3D printing with coating strategy and the guideline of combining experimental and computational analysis will disclose the relationship between the 3D structures and electrocatalytic performance and lay the foundation for the follow‐up investigation of BCH electrocatalysts.

## 2 Results and Discussion

**Scheme 1:**
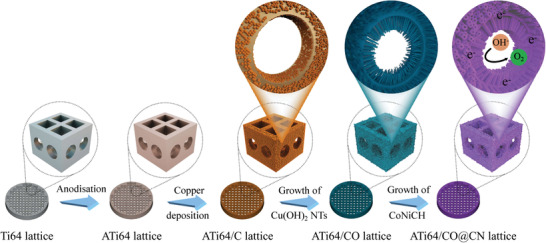
A fabrication process of the ATi64/CO@CN lattice catalyst.

The synthetic process of the anodized Ti64/Cu(OH)_2_@CoNiCH (ATi64/CO@CN) lattice catalyst is illustrated in **Scheme** **1**. First, the Ti64 lattice used for catalyst supports was manufactured by SLM 3D printing. To increase the adhesion strength of Cu coating on the surface of the Ti64 lattice, electrochemical anodization was employed to functionalize the SLM‐printed Ti64 lattice,^[^ ^15^ ^]^ which was labeled as the anodized Ti64 (ATi64) lattice. Then the electroless deposition strategy was utilized to coat the ATi64 lattice with a thin copper layer, and the ATi64/Cu lattice was labeled as the ATi64/C lattice. After the electroless deposition, the Cu(OH)_2_ nanotubes (NTs) were grown on the ATi64/C lattice, and the ATi64/Cu(OH)_2_ lattice was labeled as the ATi64/CO lattice. Finally, the ATi64/CO@CN lattice with tubular hierarchical core–shell structures was synthesized by a facile hydrothermal synthesis.^[^ ^4a^ ^]^ For comparison, the ATi64/CO@CN bulk catalyst was prepared, which adopted the same method mentioned above (Figure S1, Supporting Information). The designed Ti64 bulk and Ti64 lattice models are demonstrated in Table S1, Supporting Information.

**Figure 1:**
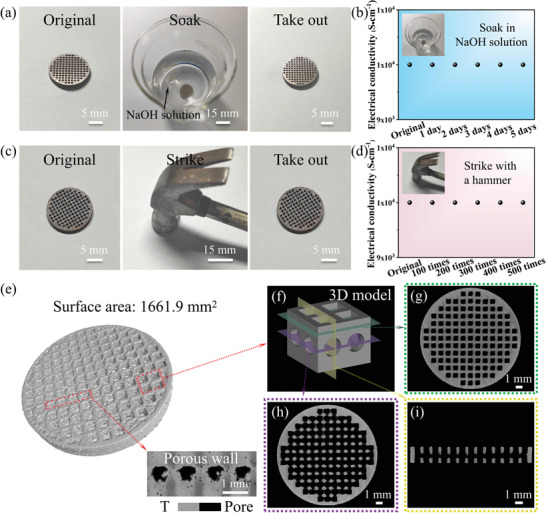
a,c) Digital photographs and b,d) electrical conductivity of the Ti64 lattice before and after soaking in 1 m NaOH solution for 5 days and striking 500 times with a hammer, respectively. e) 3D rendered image from micro‐XCT characterization. The inset figure indicates a porous wall. f) The designed 3D model unit. 2D slices of the transverse near the g) top surface and h) center surface. i) 2D slice of the transverse from the side view.

Concerning practical applications, good corrosion resistance of 3D‐printed components is vital to permanently maintain structural integrity. For OER characterization, catalysts are commonly immersed in an acid or alkaline medium.^[^ ^16^ ^]^ Therefore, obtaining corrosion‐resistant catalyst supports is a premise to guarantee the reliable performance of catalysts. **Figure** **1**a shows the corrosion test of the ATi64/CO@CN lattice in 1 m NaOH solution. After soaking in the alkali solution for 5 days and 1000 cyclic voltammetry (CV) cycles at 100 mV s^−1^ in a voltage range of 0.1–0.9 V, a negligible change of surface was found in the Ti64 lattice (Figure 1a and Figures S2 and S3a, Supporting Information). Note that the Ti64 lattice exhibited a stable electrical conductivity of 1 × 10^4^ S cm^−1^ after the corrosion studies (Figure 1b and Figure S3b, Supporting Information), suggesting outstanding corrosion resistance. In contrast, as the main catalyst support currently utilized, Cu foam is easily corroded by NaOH solution, which is not suitable for the long‐term use of OER electrocatalysts (Figure S4a, Supporting Information).

In addition to the corrosion‐resistant property, the mechanical stability of catalyst supports is another crucial characteristic for actual applications. It should be mentioned that when testing electrochemical performance, catalytic electrodes are always squeezed by electrode clips, subjected to water pressure in an aqueous solution, and even subjected to external forces during mechanical operations, bringing in a reduced lifetime. Note that Cu foam is liable to be permanently deformed by external forces, disabling the functionality of 3D conductive networks (Figure S4b, Supporting Information). Consequently, developing robust catalyst supports provides a promising strategy for increasing the service life of catalysts. Figure 1c shows the Ti64 lattice before and after striking 500 times with a hammer. It is noted that the Ti64 lattice exhibited unchanged surface morphology (Figure S5, Supporting Information) and electrical conductivity (1 × 10^4^ S cm^−1^, Figure 1d) after striking 500 times, suggesting promising mechanical stability. To accurately evaluate the mechanical property of the catalyst support, tensile tests were carried out. The model of the dog‐bone tensile specimen is displayed in Figure S6, Supporting Information, and the SLM‐printed Ti64 lattice exhibited higher mechanical strength than Cu foam (Figure S7, Supporting Information).

To investigate the internal structural characteristics of the Ti64 lattice, high‐resolution Micro X‐ray Computed Tomography (Micro‐XCT) was carried out, as shown in Figure 1e. Regarding the Ti64 lattice, a hollow porous structure with large holes was found in reconstructed 3D images. Note that the hollow porous framework with uniformly distributed and interconnected pores can be observed in typical 2D slices from the top view (Figure 1g,h) and side view (Figure 1i). In contrast, the Ti64 bulk exhibited a solid structure without any interconnected pores (Figure S8, Supporting Information). The surface area of the Ti64 lattice was 1661.9 mm^2^, which was much higher than that of the Ti64 bulk (513.2 mm^2^). Compared with the Ti64 bulk, the enhanced surface area of the Ti64 lattice will be beneficial to the larger mass loading of catalysts (exposed active sites) onto the catalyst support. Additionally, such an interconnected micro‐sized porous structure of the Ti64 lattice was designed to improve the electrolyte circulation and bubble removal, which contributed to the increased catalytic activity. The reconstructed 3D images and 2D slices of the Ti64 bulk and Ti64 lattice from micro‐XCT characterization were consistent with the designed models (Table S1, Supporting Information). Therefore, CT results showed that the catalyst support with sophisticated internal structures was successfully prepared by the SLM technology.

**Figure 2:**
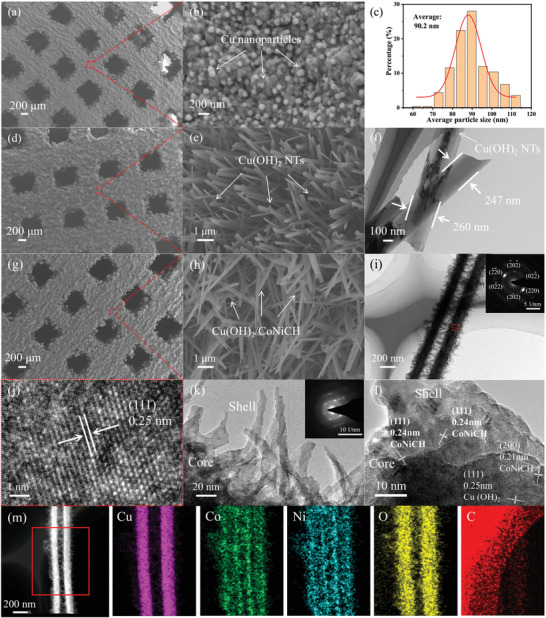
a,b) SEM images of the ATi64/C lattice. c) Particle size distribution of Cu nanoparticles coated on the ATi64/C lattice. d,e) SEM images of the ATi64/CO lattice. f) TEM image of the Cu(OH)_2_ NTs. g,h) SEM images of the ATi64/CO@CN lattice. i,k) TEM images of the Cu(OH)_2_@CoNiCH catalyst. Insets are the SAED patterns of the Cu(OH)_2_ NTs and CoNiCH nanothorns, respectively. j,l) High‐resolution TEM images of the Cu(OH)_2_ NTs and interface structure between the Cu(OH)_2_ NTs and CoNiCH, respectively. m) EDS elemental mapping of the elements of Cu, Co, Ni, O, and C.

The synthetic evolution of the Cu(OH)_2_@CoNiCH catalyst on the Ti64 lattice and Ti64 bulk is exhibited in Figure S9, Supporting Information. To explore the morphological characteristics of catalysts growing on the surface of the Ti64 lattice, scanning electron microscopy (SEM) and transmission electron microscopy (TEM) images were collected. As exhibited in Figure S10a, Supporting Information, the Ti64 lattice exhibited a rough surface, which was beneficial to the growth of catalysts.^[^ ^17^ ^]^ After the anodization, the surface morphology of the Ti64 lattice remained unchanged (Figure S10b, Supporting Information). Subsequently, Cu nanoparticles (average particle size of 90.2 nm) and Cu(OH)_2_ NTs (diameters of ≈250 nm) were densely coated on the surface of the substrate in the ATi64/C lattice (**Figure** **2**a–c) and ATi64/CO lattice (Figure 2d–f), respectively. For the ATi64/CO@CN lattice, CoNiCH nanothorns (lengths of 50–100 nm) germinated on the surface of Cu(OH)_2_ NTs (diameters of ≈300 nm) to form hollow core–shell hierarchical structures (Figure 2g–i). Such a nanoscale hierarchical structure allows fast electrolyte infiltration and ion transport, which will be beneficial to improving the catalytic performance of electrocatalysts.^[^ ^18^ ^]^ Note that the selected area electron diffraction (SAED) pattern of the Cu(OH)_2_ NTs (inset of Figure 2i) was indexed to the [111] zone axis,^[^ ^19^ ^]^ and the lattice spacing of 0.25 nm was corresponding to the (111) plane of Cu(OH)_2_ NTs (Figure 2j).^[^ ^20^ ^]^ Figure 2k,l shows the hierarchical structures containing the core of Cu(OH)_2_ NTs and shells of CoNiCH nanothorns. The SAED pattern inset of Figure 2k discloses defined diffraction rings, revealing the polycrystalline nature of CoNiCH nanothorns.^[^ ^21^ ^]^ The lattice spacing of 0.24 and 0.21 nm in Figure 2l and Figure S11, Supporting Information was associated with the (111) and (200) planes of CoNiCH nanothorns, respectively.^[^ ^5^, ^7^ ^]^ Note that the CoNiCH nanothorns were anchored on the edges of Cu(OH)_2_, and no clear interface was observed in the high‐resolution TEM characterization, suggesting the formation of a complex hierarchical structure.^[^ ^22^ ^]^

The elemental mapping of the Cu(OH)_2_@CoNiCH catalyst with hollow core–shell hierarchical structures was investigated utilizing the energy dispersive X‐ray spectrometry (EDS). Figure 2m shows the uniformly distributed elements of Cu, Co, Ni, O, and C in the backbone and branch area. Note that the element of Cu was observed only in the backbone area, which further indicated that CoNiCH nanothorns were germinated on the surface of Cu(OH)_2_ NTs.

**Figure 3:**
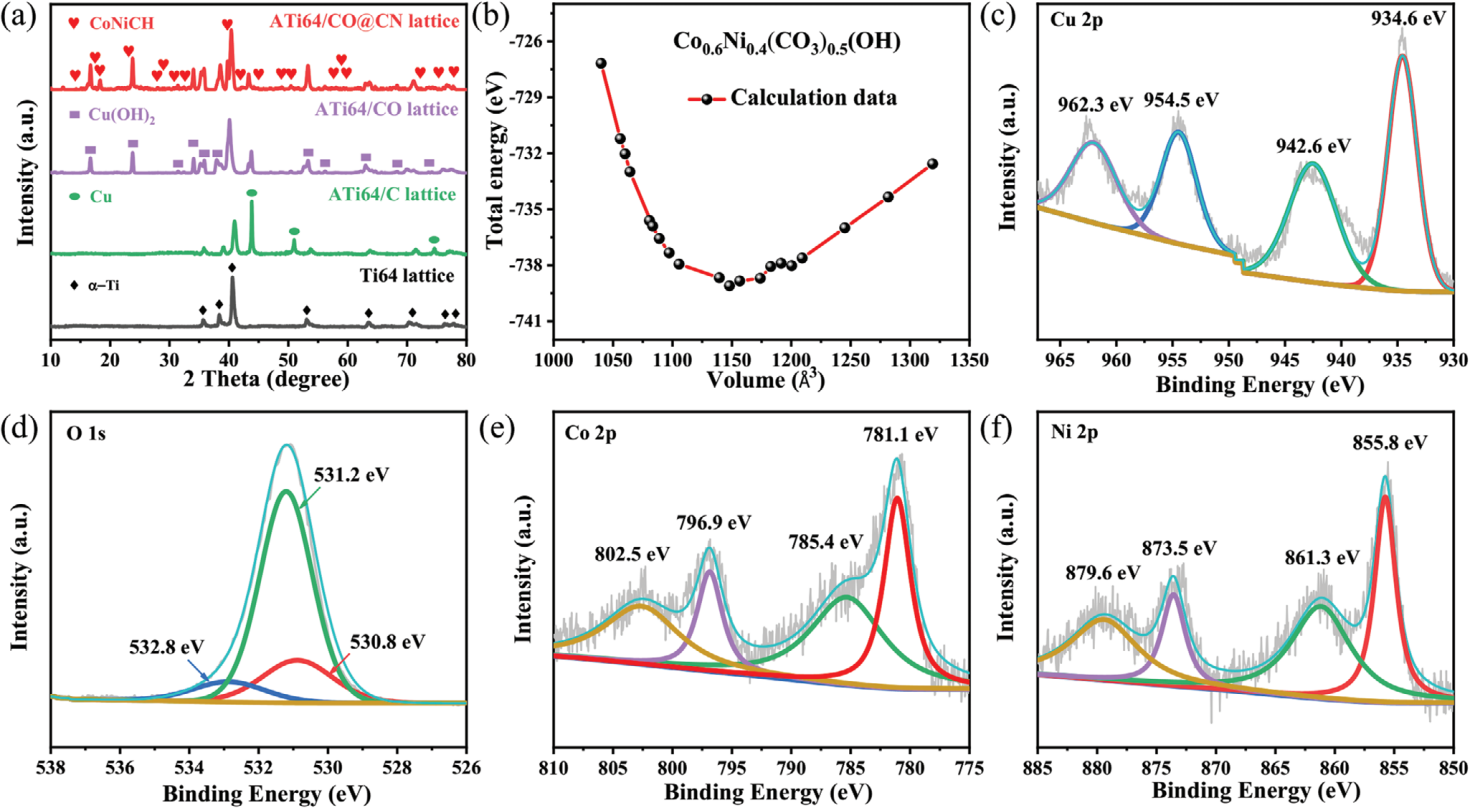
a) XRD patterns of the Ti64, ATi64/C, ATi64/CO, and ATi64/CO@CN lattice. b) Total energy plotted as a function of the volume of the unit cell of the CoNiCH nanothorns. c) Cu 2p, d) O 1s, e) Co 2p, and f) Ni 2p spectrum of the ATi64/CO@CN lattice.

To further investigate the crystallographic structure of catalysts growing on the surface of the Ti64 lattice, X‐ray diffraction (XRD) measurement was carried out. **Figure** **3**a shows XRD patterns of the Ti64, ATi64/C, ATi64/CO, and ATi64/CO@CN lattice. As exhibited in Figure 3a, the Ti64 lattice was dominated by the *α*‐Ti phase (JCPDS No. 441294) with a hexagonal close‐packed (hcp) lattice.^[^ ^23^ ^]^ After the anodization, a negligible change in crystallographic structure was noticed in the ATi64 lattice (Figure S12, Supporting Information). Compared with the Ti64 and ATi64 lattice, the ATi64/C lattice exhibited three extra peaks. Specifically, the peaks located at ≈43.8°, 51°, and 74.6° were assigned to Cu nanoparticles (JCPDS No. 85–1326), corresponding to (111), (200), and (220) lattice planes, respectively.^[^ ^24^ ^]^ For the ATi64/CO lattice, new peaks located at ≈16.7°, 23.8°, 31.5°, 34.1°, 35.9°, 38.0°, 53.2°, 56.2°, 62.9°, 68.3°, and 73.7° were attributed to orthorhombic Cu(OH)_2_ (JCPDS No. 13–0420), which was corresponding to (020), (021), (110), (002), (111), (041), (150), (151), (200), (221), and (202) lattice planes, respectively.^[^ ^25^ ^]^ Remarkably, compared with the ATi64/CO lattice, many fresh peaks appeared in the ATi64/CO@CN lattice, which was ascribed to the CoNiCH nanothorns (Figure 3a and Figure S13a, Supporting Information).

Since no standard PDF cards could match the above diffraction peaks in the ATi64/CO@CN lattice, Vienna Atomic Simulation Package (VASP) was used to clarify the specific structural parameters of the CoNiCH nanothorns. Generally, the optimized structural parameters were obtained by using the total energy minimization method.^[^ ^26^ ^]^ According to the molar ratio of Co and Ni tested by the inductively coupled plasma‐mass spectrometry (ICPMS) characterization (Table S2, Supporting Information), the total energy plotted as a function of the volume of the unit cell was computed, as shown in Figure 3b. The optimized structural parameters of the CoNiCH nanothorns were *a* = 9.6101 Å, *b* = 12.3369 Å, *c* = 9.6970 Å, *α* = *γ* = 90°, and *β* = 93.0519°, which belongs to the monoclinic system.^[^ ^27^ ^]^ The molecular formula of the CoNiCH nanothorns was Co_0.6_Ni_0.4_(CO_3_)_0.5_(OH). It should be mentioned that the calculated diffraction peaks of Co_0.6_Ni_0.4_(CO_3_)_0.5_(OH) were consistent with the XRD result of the ATi64/CO@CN lattice (Figure S13b, Supporting Information).

In addition to XRD characterization, X‐ray photoelectron spectra (XPS) measurement was also conducted to evaluate the surface electronic states of the catalysts. The XPS curves of the Ti64‐based lattice electrocatalysts were deconvoluted according to the reference of TiO_2_‐, Cu‐, CuO‐, Cu(OH)_2_‐, Co‐, and Ni‐based materials.^[^ ^28^ ^]^ The Ti64 and ATi64 lattice exhibited the same elements (Figure S14, Supporting Information). For the ATi64/C lattice, two obvious peaks at 952.1 and 932.4 eV in the Cu 2p spectrum were assigned to Cu 2p_1/2_ and Cu 2p_3/2_ of Cu^0^ (Figure S15a, Supporting Information).^[^ ^28^, ^29^ ^]^ Additionally, two peaks at 955.1 and 933.7 eV corresponded to Cu 2p_1/2_ and Cu 2p_3/2_ of Cu^2+^, and a satellite peak was observed at 944.5 eV, which was attributed to the oxidation of Cu in the air.^[^ ^28c–e^ ^]^ The O 1s spectrum (Figure S15b, Supporting Information) was divided into two peaks, in which the peak at 530.3 eV was associated with the O^2−^ from TiO_2_.^[^ ^28a,b^ ^]^ Additionally, the peak at 531.3 eV represented oxygen coordinate defects.^[^ ^30^ ^]^

Concerning the ATi64/CO and ATi64/CO@CN lattice, the Cu 2p spectrum was divided into Cu 2p_3/2_ (934.6 and 942.6 eV) and Cu 2p_1/2_ (954.5 and 962.3 eV), which were corresponding to the paramagnetic chemical state of Cu^2+^ in Cu(OH)_2_ NTs (Figure 3c and Figure S16a, Supporting Information).^[^ ^31^ ^]^ Note that a minor variation in peak positions was observed in Figure S16a, Supporting Information, which was ascribed to the interaction between the Cu(OH)_2_ NTs and CoNiCH nanothorns.^[^ ^7^ ^]^ The close interfacial contact between the Cu(OH)_2_ NTs and CoNiCH would favor enhanced charge transfer.^[^ ^32^ ^]^ Additionally, the deconvolution of the O 1s spectrum of the ATi64/CO and ATi64/CO@CN lattice was different. As illustrated in Figure S16b, Supporting Information, the O 1s spectrum of the ATi64/CO lattice was divided into two peaks (532.8 and 530.8 eV), associating with the absorbed water molecule and hydroxide group, respectively.^[^ ^5a^ ^]^ In contrast, a new peak located at 531.2 eV was noticed in the O 1s spectrum of the ATi64/CO@CN lattice (Figure 3d), corresponding to the carbonate group. This indicated that carbonate groups were formed in the CoNiCH nanothorns, which was consistent with the VASP result. As observed in Figure 3e, the Co 2p spectrum of the ATi64/CO@CN lattice was fitted and divided into Co 2p_3/2_ (781.1 eV) and Co 2p_1/2_ (796.9 eV), and two satellite peaks were found at 785.4 and 802.5 eV, indicative of the Co^2+^ in the CoNiCH nanothorns.^[^ ^28^, ^33^ ^]^ Additionally, the peaks at 855.8 and 873.5 eV in the Ni 2p spectrum of the ATi64/CO@CN lattice were attributed to Ni 2p_3/2_ and Ni 2p_1/2_, and two satellite peaks were observed at 861.3 and 879.6 eV, which indicated the Ni^2+^ in the CoNiCH nanothorns (Figure 3f).^[^ ^28^, ^34^ ^]^ These characteristics provide abundant evidence for verifying the formation of CoNiCH in the ATi64/CO@CN lattice.

**Figure 4:**
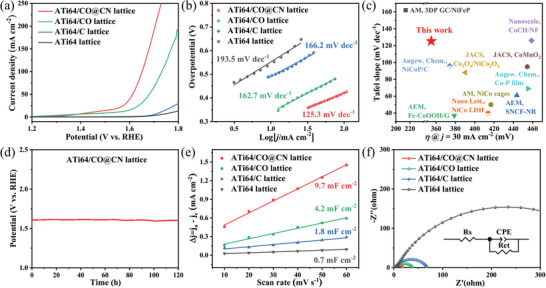
a) Polarization curves and b) Tafel plots of the ATi64, ATi64/C, ATi64/CO, and ATi64/CO@CN lattice. c) Comparison of the overpotential (at 30 mA cm^−2^) and Tafel slope of the present work with other transition metal‐based electrocatalysts reported in the literature.^[^ ^35^, ^44^ ^]^ d) The durability test of the ATi64/CO@CN lattice at 30 mA cm^−2^ for 120 h. e) Current density differences plotted as a function of the scan rates and f) Nyquist plots of the ATi64, ATi64/C, ATi64/CO, and ATi64/CO@CN lattice. The inset is a simulated equivalent circuit.

Having a detailed characterization of the morphology and structure of the as‐prepared catalysts, the following vital point is their electrochemical performance. The catalytic activities of various electrodes were evaluated utilizing a typical three‐electrode system. **Figure** **4** shows the corresponding electrochemical performance of the as‐prepared ATi64, ATi64/C, ATi64/CO, and ATi64/CO@CN lattice. As shown in Figure 4a, the ATi64 and ATi64/C lattice exhibited negligible OER activity. After the growth of Cu(OH)_2_ NTs, the OER activity of the ATi64/CO lattice increased due to the large surface area and active sites resulting from the tubular open structure of Cu(OH)_2_.^[^ ^7^ ^]^ Compared with the ATi64/CO lattice, the ATi64/CO@CN lattice displayed a higher electrocatalytic activity (Figure 4a). This implied that the growth of CoNiCH nanothorns further increased the exposed active sites, contributing to an enhanced electrocatalytic effect on OER performance.^[^ ^7^ ^]^ Remarkably, the ATi64/CO@CN lattice showed overpotentials of 355 and 382 mV at various current densities of 30 and 50 mA cm^−2^, which outperformed the ATi64/CO lattice, ATi64/C lattice, and ATi64 lattice, respectively (Table S3, Supporting Information). In addition to the nanoscale hierarchical core–shell structure of the Cu(OH)_2_@CoNiCH catalyst, the structure of catalyst supports also played an important role in electrocatalytic performance. As exhibited in Figure S17, Supporting Information, the ATi64 and ATi64/CO@CN lattice delivered higher electrocatalytic activities than the ATi64 and ATi64/CO@CN bulk, respectively. This verified that the existence of hollow porous structures not only increased the active sites of catalysts but also improved the bubble escaping and electrolyte exchange during the OER process.^[^ ^9^ ^]^ Therefore, the unique micro/nano‐sized hierarchical porous structures, including the microscale hollow structure of the catalyst support and nanoscale core–shell porous structure of the catalyst, contributed to the improved electrocatalytic performance of the ATi64/CO@CN lattice.

To explore the OER kinetics of the catalytic electrodes, the corresponding Tafel plots were carried out. Generally, a low Tafel slope represents rapid reaction kinetics, resulting in high electrocatalytic activity for OER.^[^ ^35^ ^]^ As shown in Figure 4b, the Tafel slope of the ATi64/CO@CN lattice was 125.3 mV dec^−1^, which was lower than that of the ATi64 lattice (193.5 mV dec^−1^), ATi64/C lattice (166.2 mV dec^−1^), and ATi64/CO lattice (162.7 mV dec^−1^), indicating the fast kinetics for OER.^[^ ^7^ ^]^ The low overpotential and Tafel slope of the ATi64/CO@CN lattice are comparable to the most transition metal‐based electrocatalysts reported in the literature (Figure 4c). The electrochemical stability of the Cu(OH)_2_@CoNiCH catalyst is another critical attribute for OER catalysts. As observed in Figure 4d, the chronopotentiometry characterization indicated that a negligible potential change was observed in the ATi64/CO@CN lattice electrode at a current density of 30 mA cm^−2^ for 120 h, which suggested stable electrocatalytic activity for the long‐term application. In addition to the electrochemical stability, the ATi64/CO@CN lattice exhibited promising mechanical stability. As exhibited in Figure S18, Supporting Information, almost unchanged catalytic activity was observed after the 100th clamping/detaching cycle. To explore the morphology and composition of the ATi64/CO@CN lattice after the stability test, XRD, SEM, and XPS measurements were carried out. The XRD exhibited that no obvious change was observed in the position of the diffraction peaks of the CoNiCH before and after the stability test (Figure S19, Supporting Information). As shown in Figure S20, Supporting Information, the tubular hierarchical core–shell structure of the catalyst was slightly agglomerated after a long cycle reaction. Regarding the ATi64/CO@CN lattice after the stability test, a new peak located at ≈780 eV was noticed in the Co 2p spectrum, which was ascribed to the Co^3+^ from CoOOH (Figure S21a, Supporting Information).^[^ ^5a^ ^]^ Additionally, a new peak located at ≈857 eV in Figure S21b, Supporting Information, indicated the formation of NiOOH,^[^ ^36^ ^]^ which implied a small amount of oxidation of CoNiCH to CoNi oxyhydroxide.

Remarkably, the electrochemically active surface areas (ECSAs) of the ATi64, ATi64/C, ATi64/CO, and ATi64/CO@CN lattice were estimated from the capacitance of the double‐layer capacitor (*C*_dl_) according to the CV curves (Figure S22, Supporting Information). The *C*_dl_ of the ATi64/CO@CN lattice was 9.7 mF cm^−2^, which was higher than 0.7 mF cm^−2^ of the ATi64 lattice, 1.8 mF cm^−2^ of the ATi64/C lattice, and 4.2 mF cm^−2^ of the ATi64/CO lattice (Figure 4e). The enhanced ECSAs can be ascribed to the highly exposed active sites provided by Cu(OH)_2_ NTs and CoNiCH nanothorns. More importantly, compared with the ATi64/CO@CN bulk (2 mF cm^−2^), the *C*_dl_ of the ATi64/CO@CN lattice increased by ≈4 times, indicating the positive effect of architecture design on ECSAs of electrodes (Figure S23, Supporting Information). Note that turnover frequency (TOF) is used to characterize the intrinsic catalytic activity of OER electrocatalysts. The TOF value (s^−1^) is obtained by employing the equation:

(1) $$\mathrm{TOF}=A\times j/\left(4\times F\times N\right)$$

where *A* signifies the surface area of the electrode, *j* indicates the current density at a certain overpotential, *F* denotes the Faraday constant, and *N* indicates the number of active sites on the electrode.^[^ ^7^ ^]^ The TOF value of the ATi64/CO@CN lattice was 0.0179 s^−1^ (*η* = 500 mV), which was higher than that of the ATi64/C (0.0002 s^−1^, *η* = 500 mV) and ATi64/CO (0.0021 s^−1^, *η* = 500 mV), indicating that the ATi64/CO@CN exhibited much higher intrinsic activity. The high TOF values of the ATi64/CO@CN lattice (0.0012 s^−1^, *η* = 300 mV; 0.0050 s^−1^, *η* = 400 mV; 0.0179 s^−1^, *η* = 500 mV) were comparable to those of other metal‐based electrocatalysts reported in the literature, including Co_3_S_4_/TETA (0.00132 s^−1^, *η* = 500 mV),^[^ ^37^ ^]^ Co‐Bi film (0.0015 s^−1^, *η* = 400 mV),^[^ ^38^ ^]^ CoO‐cal (0.0013 s^−1^, *η* = 300 mV),^[^ ^39^ ^]^ NiCo@NCNTs‐600 (0.000662 s^−1^, *η* = 440 mV),^[^ ^40^ ^]^ Co‐Pi film (0.002 s^−1^, *η* = 410 mV),^[^ ^41^ ^]^ and CS‐NFO@PNC‐900 (0.00085 s^−1^, *η* = 270 mV).^[^ ^42^ ^]^ To investigate the reaction kinetics of various electrodes, the electrochemical impedance spectra (EIS) of the ATi64, ATi64/C, ATi64/CO, and ATi64/CO@CN lattice were recorded, and the corresponding Nyquist plots are exhibited in Figure 4f. Additionally, an equivalent circuit (inset of Figure 4f) was simulated, where Rs corresponds to a solution resistance, CPE denotes a constant phase element, and Rct indicates a charge transfer resistance, respectively. As shown in Table S4, Supporting Information, the Rct of the ATi64/CO@CN lattice (15.8 Ω) was smaller than that of the ATi64 lattice (453.7 Ω), ATi64/C lattice (65.9 Ω), and ATi64/CO lattice (36.3 Ω), indicating a faster charge transfer rate for OER in the ATi64/CO@CN lattice.^[^ ^43^ ^]^

**Figure 5:**
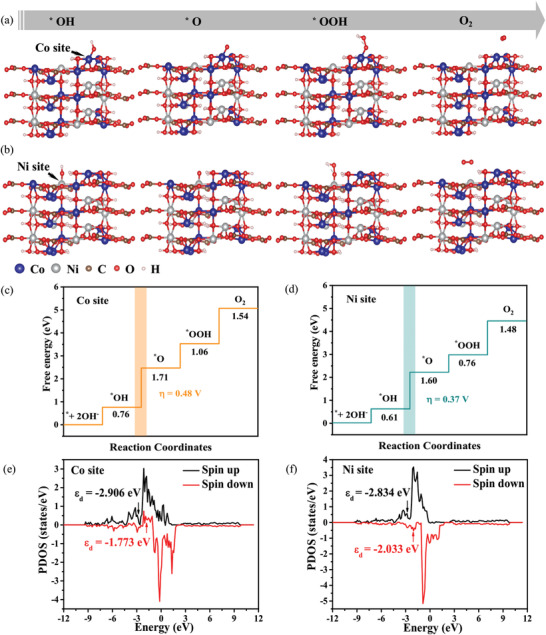
Molecular structures of the *OH, *O, *OOH, and O_2_ intermediates at the a) Co and b) Ni sites of the CoNiCH. Gibbs free energy graphs of OER and *ε*_d_ of the CoNiCH with c,e) Co and d,f) Ni sites, respectively.

To gain an insight into the origin of the promising catalytic performance of the ATi64/CO@CN lattice for OER, DFT calculations were performed. Since the catalyst was composed of Cu(OH)_2_ NTs and CoNiCH nanothorns, atomic configurations of the Cu(OH)_2_ and Co_0.6_Ni_0.4_(CO_3_)_0.5_(OH) were constructed (Figure S24, Supporting Information). The OER active sites of Cu(OH)_2_ and CoNiCH were designated as the Cu atom and Co/Ni atoms, respectively. Generally, it is assumed that the OER mechanism performs a four‐step reaction through the intermediates of *OH, *O, *OOH, and O_2_ in an alkaline solution (**Figures** **5**a,b for the CoNiCH with Co and Ni sites, respectively).^[^ ^45^ ^]^ For Cu(OH)_2_, various intermediates during the OER process are illustrated in Figure S25a, Supporting Information. As illustrated in Figures 5c,d, the rate‐determining step of the OER process at the Co and Ni sites of the CoNiCH was the *O formation step, with a binding energy of 1.71 and 1.60 eV, respectively. Additionally, the binding energies of the *OH, *OOH, and O_2_ at the Co/Ni sites of the CoNiCH were 0.76/0.61, 1.06/0.76, and 1.54/1.48 eV, respectively. Concerning the first‐step reaction to form the *OH species, the Ni site of the CoNiCH exhibited lower binding energy than the Co site, which implied that the Ni active site was easier to adsorb OH^−^. Similarly, the rate‐determining step for the Cu(OH)_2_ with Cu site was the *O formation step with a binding energy of 1.97 eV, which was higher than the CoNiCH with Co or Ni site (Figure S25b, Supporting Information). The theoretical overpotentials (*η*) were computed according to the equation:^[^ ^46^ ^]^

(2) $$\eta =\max\left[\Delta G1,\Delta G2,\Delta G3,\Delta G4\right]/e-1.23$$

where △*G*1, △*G*2, △*G*3, and △*G*4 indicate the Gibbs free energy of the formation of intermediates of *OH, *O, *OOH, and O_2_, respectively. The *η* of the CoNiCH with Co site, CoNiCH with Ni site, and Cu(OH)_2_ with Cu site were 0.48, 0.37, and 0.74 V, respectively (Figures 5c,d, respectively, and Figure S25b, Supporting Information). Therefore, regarding the active material level, the promising electrocatalytic performance of the ATi64/CO@CN lattice was dominated by the CoNiCH, and the Ni active site in the CoNiCH can regulate the binding energies of various intermediates to reach an optimal value, which effectively improved the electrocatalytic activity for OER. It should be mentioned that NiCo‐based materials typically undergo structural reconstruction and form the real catalytic surface of NiCo oxyhydroxides. The corresponding various intermediates during the OER process and Gibbs free energy graphs of the CoOOH and NiOOH were also investigated. As shown in Figure S26b,d, Supporting Information, the rate‐determining step for the CoOOH with Co site and NiOOH with Ni site were the same as the CoNiCH (*O formation step), with a binding energy of 1.86 and 1.74 eV, respectively. The calculated theoretical overpotential of the CoOOH and NiOOH was 0.63 and 0.51 V, which was higher than that of the CoNiCH (0.48 V at the Co site and 0.37 V at the Ni site). However, only a small amount of oxidation of CoNiCH to CoNi oxyhydroxide occurred during the OER process (Figure S21, Supporting Information), which could be negligible for the OER catalysis. Therefore, the theoretical calculation of the CoNiCH provided valuable information for understanding OER catalysis.

To disclose the synergistic impact of Co and Ni on the electrocatalytic performance of the CoNiCH, the atomic configurations, corresponding various intermediates during the OER process, and Gibbs free energy graphs of the CoCH (Co(CO_3_)_0.5_(OH)) and NiCH (Ni(CO_3_)_0.5_(OH)) were also constructed (Figure S27, Supporting Information). Note that the Co and Ni atoms were chosen as the active sites for the CoCH and NiCH, respectively. Remarkably, the rate‐determining step for the CoCH with Co site and NiCH with Ni site was the same as the CoNiCH (*O formation step), with a binding energy of 1.89 and 1.76 eV, respectively. The calculated theoretical overpotential of the CoCH and NiCH was 0.66 and 0.53 V, which was higher than that of the CoNiCH (0.48 V at the Co site and 0.37 V at the Ni site). This indicated that the introduction of a second transition metal can effectively improve the OER performance of electrocatalysts compared with a single‐metal system.^[^ ^46^ ^]^ Furthermore, the d‐band center (*ε*_d_) was carried out to acquire the positive role of catalytic activities of various active sites.^[^ ^45^ ^]^ Based on the d‐band theory, *ε*_d_, which is closer to the Fermi level, will result in stronger binding strength between the active site and adsorbates and lead to higher overpotentials.^[^ ^47^ ^]^ Herein, the *ε*_d_↑ (electron spin up) and *ε*_d_↓ (electron spin down) values of the Ni site were −2.834 and −2.033 eV, while those of the Co site were −2.906 and −1.773 eV, respectively (Figures 5e,f, respectively). The *ε*_d_↑ of the Ni site was close to that of the Co site, while the *ε*_d_↓ of the Ni site is lower than that of the Co site, which suggested the d‐band of Ni is far away from the Fermi level, thus the H_2_O evolution overpotential of the Ni site is lower than Co site. Additionally, an energy band gap (*E*_g_) was used to evaluate the electronic conductivity of the catalytic electrodes.^[^ ^48^ ^]^ As shown in Figure S28, Supporting Information, the CoNiCH (0.663 eV) exhibited a lower *E*_g_ than the Cu(OH)_2_ (0.899 eV), CoCH (1.623 eV), and NiCH (1.352 eV), displaying an essence of optimal electron transfer in the CoNiCH. Therefore, the DFT analysis provided an essential comprehension of the CoNiCH as a high‐performance electrocatalyst for OER.

## 3 Conclusion

In this study, we have designed and prepared a novel Ti–6Al–4V titanium alloy (Ti64) lattice catalyst support for OER through the SLM 3D printing technology. Remarkably, the Ti64 lattice exhibited several advantageous characteristics such as good corrosion resistance in alkaline media, excellent mechanical stability, promising electrical conductivity, and a large surface area. After coating the highly active Cu(OH)_2_@CoNiCH catalyst on the surface of the Ti64 lattice, a unique micro/nano‐sized hierarchical porous structure was constructed, including microscale holes formed by structural design and nanoscale holes from the core–shell Cu(OH)_2_@CoNiCH catalyst, which all contributed to the enhanced electrocatalytic activity. Remarkably, a promising electrocatalytic performance with a low overpotential of 355 mV at 30 mA cm^−2^ and a Tafel slope of 125.3 mV dec^−1^ was achieved. The DFT analysis demonstrated that the CoNiCH nanothorns mainly contributed to the increased OER performance, and the Ni active site of the CoNiCH provided a faster *O intermediate adsorption than the Co site, helping to speed up the entire redox reactions. The DOS calculation also confirmed the positive effect of the Ni site in CoNiCH and optimized electron transfer of the CoNiCH with a diatomic doping strategy. This study presents a novel insight into exploiting alternative catalyst supports and exploring the intrinsic activities for OER, which provided an instructive strategy for next‐generation OER electrocatalysts. Ti64 catalyst supports have promising mechanical stability and corrosion resistance, which can be used for the electrolysis of industrial wastewater to produce hydrogen. Additionally, 3D printing technology can be utilized to design a variety of substrate structures that suit various applications. In the future, more 3D‐printed electrodes will be introduced in the applications of electrocatalysis, photocatalysis, batteries, etc., contributing to clean energy and carbon neutrality.

## 4 Experimental Section

### Materials

Spherical Ti64 powders with a size range of 15–53 µm, supplied by Oerlikon company (Switzerland), were used to fabricate Ti64 catalyst supports. Colloidal palladium activator, sodium chloride (NaCl, purity ≥99.5%), and hydrochloric acid (HCl, purity: 36–38 wt%) were manufactured by Jixin Chemical Technology Co. Ltd. (Guangzhou, China), Xilong Science Co., Ltd (Shantou, China), and Dongjiang Chemical Reagent Co., Ltd (Dongguan, China), respectively. Copper sulfate (CuSO_4_, purity ≥99%) and edetate disodium (EDTA 2Na, purity ≥99.5%) were manufactured by Sigma Aldrich (America) and Dibai Biological Technology Co., Ltd (Shanghai, China), respectively. Sodium hydroxide (NaOH, purity: 99.9%), Potassium ferrocyanide trihydrate (K_4_FeC_6_N_6_ 3H_2_O, purity: 98%), Potassium sodium tartrate tetrahydrate (C_4_H_4_O_6_KNa 4H_2_O, purity: 99%), formaldehyde solution (HCHO, purity: 37 wt%), ammonium persulfate ((NH_4_)_2_S_2_O_8_, purity ≥98%), cobalt sulfate heptahydrate (CoSO_4_ 7H_2_O, purity ≥99%), nickel sulfate hexahydrate (NiSO_4_ 6H_2_O, purity: 99.9%), and CO(NH_2_)_2_ (purity ≥99.5%) were acquired from Aladdin Chemical Co., Ltd (Shanghai, China). Deionized (DI) water was acquired utilizing a water purification system (Mini‐Q Water). All chemical reagents were directly utilized without any purification.

### Preparation of 3D Ti64 Catalyst Supports

Ti64 catalyst supports with bulk and lattice structures were drawn utilizing SolidWorks software. The SLM 3D printing was performed on SLM DiMetal‐100H equipment (Laseradd Technology Co. Ltd., Guangzhou, China). The layer thickness, hatch spacing, scanning speed, and laser power in the printing were set at 0.03 mm, 0.065 mm, 1100 mm s^−1^, and 170 W, respectively.

### Anodization of Ti64 (ATi64) Lattice

The anodization was performed in the 1 m NaOH electrolyte at 13 V for 60 s under a two‐electrode system utilizing a dual output DC power supply (Keysight E3649A), in which an SLM‐printed Ti64 lattice and a platinum foil (20 × 20 mm) were utilized as the working electrode and counter electrode, respectively. After the functionalization, the Ti64 lattice was extracted, washed with DI water, and further dried at 30 °C for 24 h. The resulting sample was labeled as an ATi64 lattice. For further activation, the ATi64 lattice was immersed in 100 mL diluted colloid palladium solution consisting of 3 mL colloid palladium solution, 18 g NaCl, 1.5 mL HCl, and DI water for 12 h.

### Preparation of ATi64/Cu (ATi64/C) Lattice

A 20 mL aqueous solution containing 9–10 g L^−1^ CuSO_4_, 20–22 g L^−1^ EDTA 2Na, 14–16 g L^−1^ NaOH, 5–10 mg L^−1^ K_4_FeC_6_N_6_ 3H_2_O, 14–18 g L^−1^ C_4_H_4_O_6_KNa 4H_2_O, and 40–45 g L^−1^ HCHO was prepared for electroless copper deposition.^[^ ^49^ ^]^ The activated ATi64 lattice was immersed in the aqueous solution for 0.5 h. After the copper deposition, the lattice was extracted, washed with DI water, and further dried at 30 °C for 24 h. The resulting sample was labeled as ATi64/C lattice, and the mass loading of Cu nanoparticles on the ATi64 lattice was ≈7.99 mg cm^−2^.

### Preparation of ATi64/Cu(OH)_2_ (ATi64/CO) Lattice

A 15 mL aqueous solution containing 4 mL NaOH (10 m), 2 mL (NH_4_)_2_(S_2_O_8_) (1 m), and 9 mL DI water was prepared to synthesize Cu(OH)_2_ nanotubes.^[^ ^50^ ^]^ The ATi64/C lattice was put in the above‐mixed solution for 20 min. Subsequently, the lattice was extracted, washed with DI water, and further dried at 30 °C for 24 h. The resulting sample was labeled as ATi64/CO lattice, and the mass loading of Cu(OH)_2_ NTs on the ATi64 lattice was ≈11.75 mg cm^−2^.

### Preparation of ATi64/Cu(OH)_2_@CoNiCH (ATi64/CO@CN) Lattice

First, a 40 mL aqueous solution containing 0.28 g CoSO_4_ 7H_2_O, 0.1 g NiSO_4_ 7H_2_O, and 0.36 g urea was prepared. Subsequently, the above‐mixed solution was moved to a 50 mL polytetrafluoroethylene (PTFE)‐lined autoclave, and the as‐prepared ATi64/CO lattice was put into the PTFE lining. After the reaction for 3 h at 85 °C, the lattice was extracted, washed with DI water, and further dried at 30 °C for 24 h. The final sample was labeled as ATi64/CO@CN lattice, and the mass loading of Cu(OH)_2_@CoNiCH on the ATi64 lattice was measured to be ≈12.37 mg cm^−2^. For comparison, the ATi64/CO@CN bulk catalyst was prepared by adopting the same method mentioned above, and the mass loading of Cu(OH)_2_@CoNiCH on the ATi64 bulk was ≈3.17 mg cm^−2^.

### Characterization

The morphological characterization was carried out on field emission scanning electron microscopy (FESEM, ZEISS Merli, Germany) and TEM (300 kV, FEI Tecnai G^2^ F30, America). The EDS was collected on a TEM (FEI Tecnai G^2^ Spirit, America) at 120 kV. The electrical conductivity of the SLM‐printed Ti64 lattice was recorded on a four‐point probe (RTS‐8) technique. The tensile tests were carried out utilizing a universal testing machine (TSE504C, Shenzhen, China) at a strain rate of 5 mm min^−1^. Micro‐XCT characterization was conducted on a diondo d2 (ND Inspection & Control Solution, China), and the VG software was employed to create the images of SLM‐printed parts. XRD characterization was conducted on a Rigaku SmartLab diffractometer (Rigaku, Japan) at a scanning rate of 2° from 10° to 80°. XPS was obtained on a Thermo Scientific K‐ALPHA (England). The binding energies of the samples were calibrated concerning the C 1s peak by the carbon tape at 284.8 eV.^[^ ^51^ ^]^ The Co, Ni, and Cu molar ratios of the ATi64/CO@CN lattice were confirmed by ICPMS (Agilent 7700, Japan).

### Electrochemical Measurements

Electrochemical characterizations were carried out utilizing a CHI660D electrochemical workstation (CHI Instruments Inc., Shanghai, China). A standard three‐electrode system was adopted, in which an ATi64/CO@CN lattice, a platinum plate (15 mm × 15 mm), and an Ag/AgCl (3.5 m KCl) were utilized as the working, counter, and reference electrode, respectively. To correct the Ohmic drop of the solution, linear sweep voltammetry (LSV) measurement was carried out with a 90% iR compensation utilizing a scanning speed of 10 mV s^−1^ in 100 mL NaOH (1 m) solution. Chronopotentiometry was performed at 30 mA cm^−2^ for 120 h. For the corrosion test of the Ti64 lattice and Cu foam, continuous CV cycling in a voltage range of 0.1–0.9 V versus Ag/AgCl at 100 mV s^−1^ was performed in the 100 mL NaOH (1 m) solution. For the measurement of the capacitance of the double‐layer capacitor (*C*_dl_), CV characterization was performed in a voltage range of 0.02–0.12 V versus Ag/AgCl with various scanning rates in the 100 mL NaOH (1 m) solution. The EIS was collected using a frequency range of 0.01–100 000 Hz at a sinusoidal voltage amplitude of 5 mV. All the measured potentials were calibrated against a reversible hydrogen electrode (RHE) employing the Nernst equation:

(3) $$E\left(\mathrm{RHE}\right)=E\left(\mathrm{Ag}/\mathrm{AgCl}\right)+0.059pH+0.197$$

The overpotential (*η*) was obtained by employing the following equation:

(4) $$\eta =E\left(\mathrm{RHE}\right)-1.23$$

### Computational Details

The DFT calculation was performed on the VASP code. [Co_0.6_Ni_0.4_(CO_3_)_0.5_(OH)]_24_ and [Cu(OH)_2_]_8_ supercells were constructed according to the previously reported structures.^[^ ^52^ ^]^ To reveal the synergistic impact of Co and Ni on the electrocatalytic performance of the CoNiCH, [Ni(CO_3_)_0.5_(OH)]_24_, and [Co(CO_3_)_0.5_(OH)]_24_ supercells were also constructed to perform the calculation. Perdew–Burke–Ernzerhof (PBE) exchange‐correlation functional was employed to describe electron–orbit interaction energy.^[^ ^53^ ^]^ The cutoff energy was set to 500 eV, and k‐point sampling was selected in the first Brillouin zone with an energy tolerance of 10^−5^ eV per atom. During the geometric optimization, the lattice constants and atomic positions were relaxed, and the electronic minimization accuracy was 1 × 10^−5^ eV. The LDA + U correction calculation was used for Ni, Co, and Cu atoms, in which *U* = 4.216 eV and *J* = 0.816 eV for Ni atoms, *U* = 4.216 eV and *J* = 0.8024 eV for Co atoms, *U* = 4.080 eV and *J* = 0.789 eV for Cu atoms, respectively.^[^ ^54^ ^]^

## Conflict of Interest

The authors declare no conflict of interest.

## Supporting information

Supporting Information

## Data Availability

Research data are not shared.
