# Supplementary material for: 3D Printing of Multiscale Ti64‐Based Lattice Electrocatalysts for Robust Oxygen Evolution Reaction
Source: Adv Sci (Weinh). 2022 Jul 20;9(24):2201751. doi: 10.1002/advs.202201751 (PMC9405505; doi:10.1002/advs.202201751)
Supplement: Supplementary file 1 — Supporting Information [file ADVS-9-2201751-s001.pdf]

ADVANCED SCIENCE
Open Access

Supporting Information

for *Adv. Sci.*, DOI 10.1002/advs.202201751

3D Printing of Multiscale Ti64-Based Lattice Electrocatalysts for Robust Oxygen Evolution Reaction

Binbin Guo, Jiahui Kang, Tianbiao Zeng, Hongqiao Qu, Shixiang Yu, Hui Deng and Jiaming Bai**

3D printing of multiscale Ti64-based lattice electrocatalysts for robust oxygen evolution reaction

Binbin Guo, Jiahui Kang, Tianbiao Zeng, Hongqiao Qu, Shixiang Yu, Hui Deng, and Jiaming Bai**

Mr. B.B. Guo, Mrs. J.H. Kang, Dr. T.B. Zeng, Mr. H.Q. Qu, Mr. S.X. Yu, Prof. H. Deng, Prof. J.M. Bai

Mr. B.B. Guo, Mrs. J.H. Kang, Mr. H.Q. Qu, Mr. S.X. Yu, Prof. H. Deng, Prof. J.M. Bai
Department of Mechanical and Energy Engineering, Southern University of Science and Technology, Shenzhen, 518055, China
Email: baijm@sustech.edu.cn, dengh@sustech.edu.cn.

Dr. T.B. Zeng
School of Chemistry and Materials Engineering, Wenzhou University, Wenzhou 325035, PR China

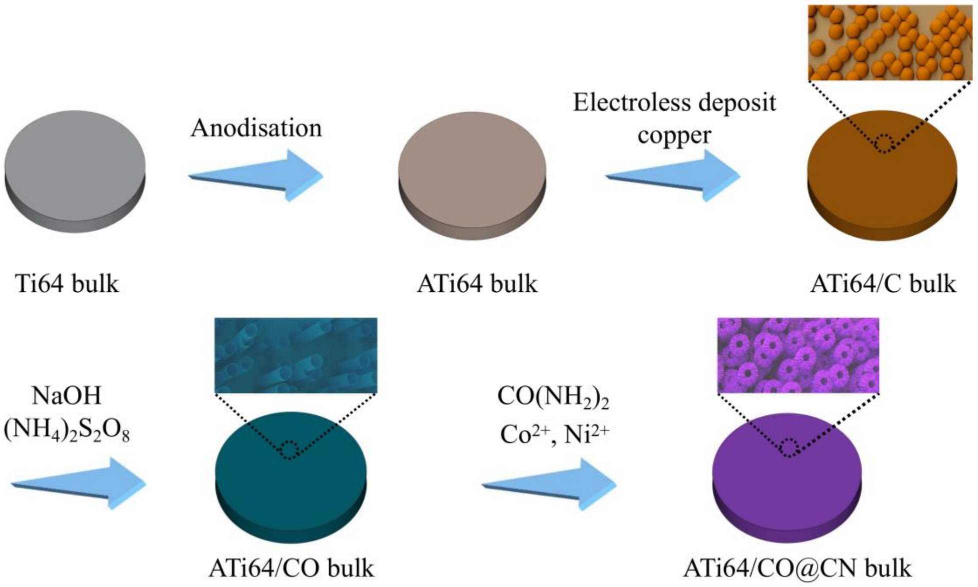

Figure S1. A fabrication process of the ATi64/CO@CN bulk catalyst.

Table S1. List of designed catalyst supports prepared by SLM 3D printing.

**Sample name**	**Sample description**	**Sample geometry**	**Design dimensions**	**Model surface area (mm^2)**
**Ti64 bulk**	3D printed solid circular plate	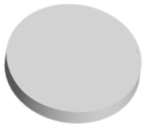	Circular plate dimensions: 15 mm (diameter) and 2 mm (height)	447.0
**Ti64 lattice**	Hollow porous circular lattice	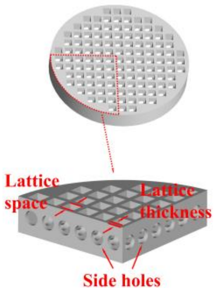	Circular structure dimensions: 15 mm (diameter) and 2 mm (height)<br>Lattice space: 0.8 mm<br>Lattice thickness: 0.3 mm<br>Diameter of side holes: 0.8 mm	1013.3

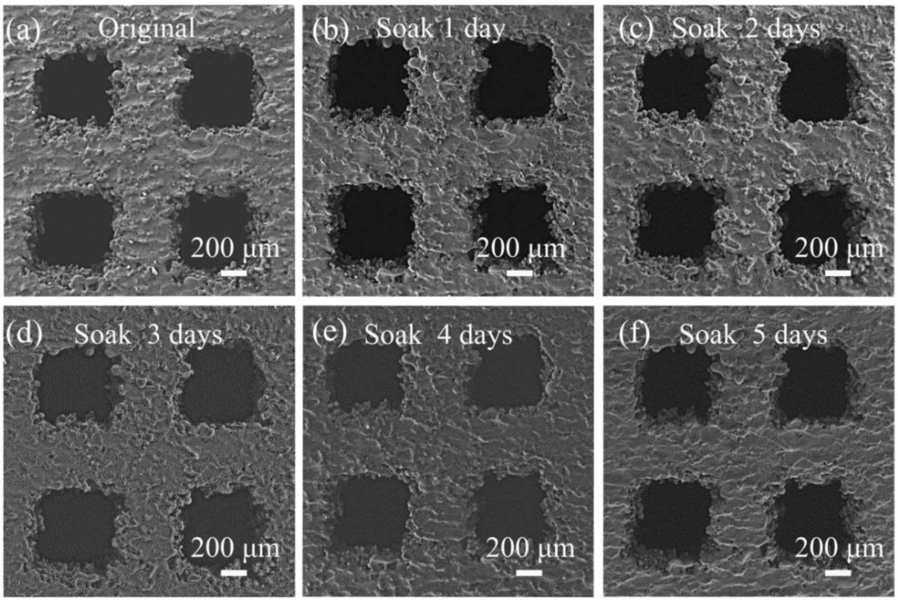

Figure S2. SEM images of Ti64 lattice a) before and after soaking in 1 M NaOH solution for b) 1 day, c) 2 days, d) 3 days, e) 4 days, and f) 5 days.

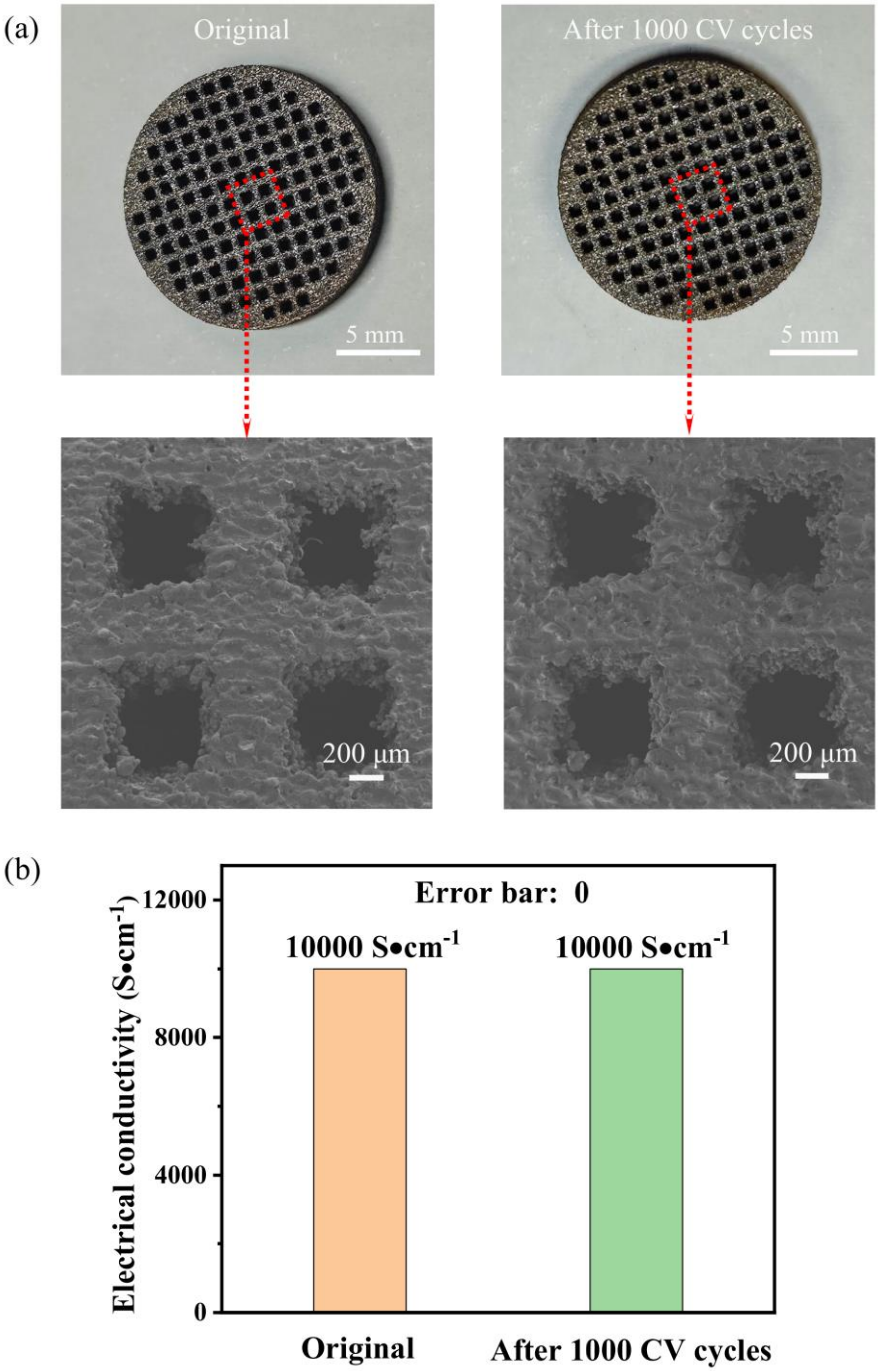

Figure S3. a) Digital photographs, SEM images, and b) electrical conductivity of the Ti64 lattice before and after 1000 CV cycles at 100 mV s^{-1} in a voltage range of 0.1-0.9 V in 1 M NaOH.

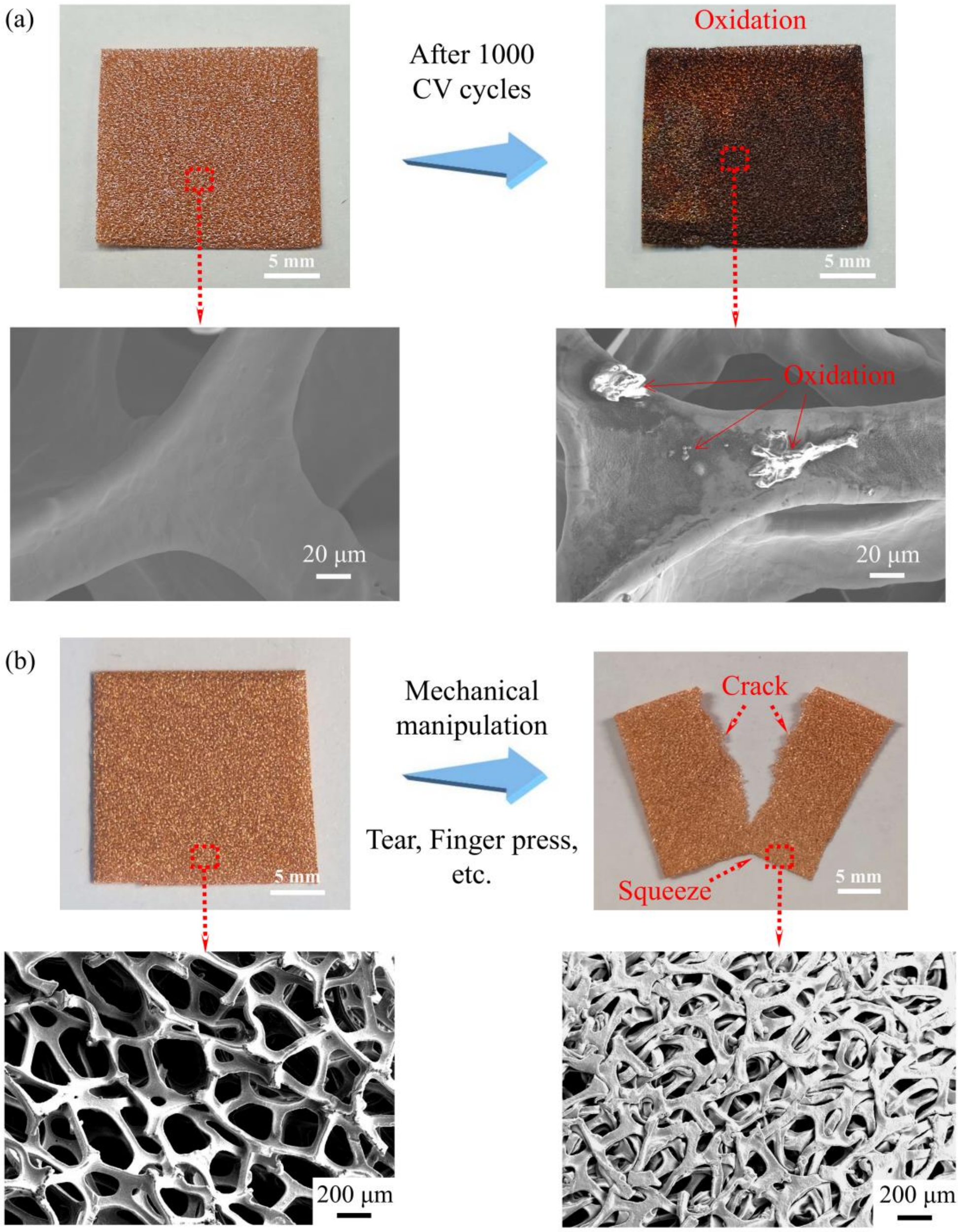

Figure S4. a) Digital photographs and SEM images of the Cu foam before and after 1000 CV cycles at 100 mV s^{-1} in a voltage range of 0.1-0.9 V in 1 M NaOH. b) Digital photographs and SEM images of the Cu foam before and after mechanical manipulation, such as tearing and finger press. The copper foam exhibited inferior corrosion-resistance and mechanical stability.

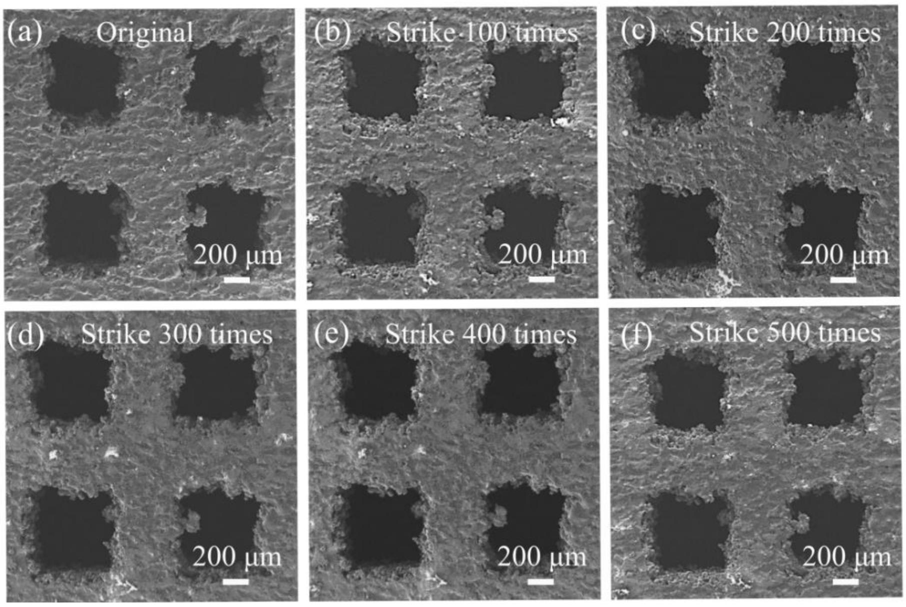

Figure S5. SEM images of Ti64 lattice a) before and after striking b) 100 times, c) 200 times, d) 300 times, e) 400 times, and f) 500 times with a hammer.

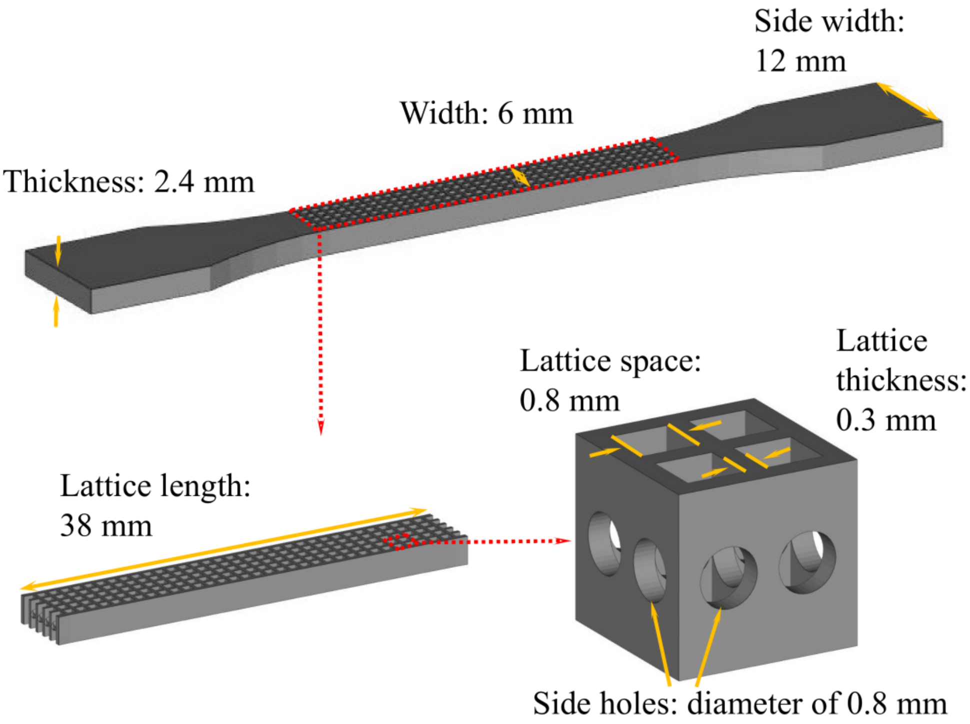

Figure S6. The tensile test model of the Ti64 lattice.

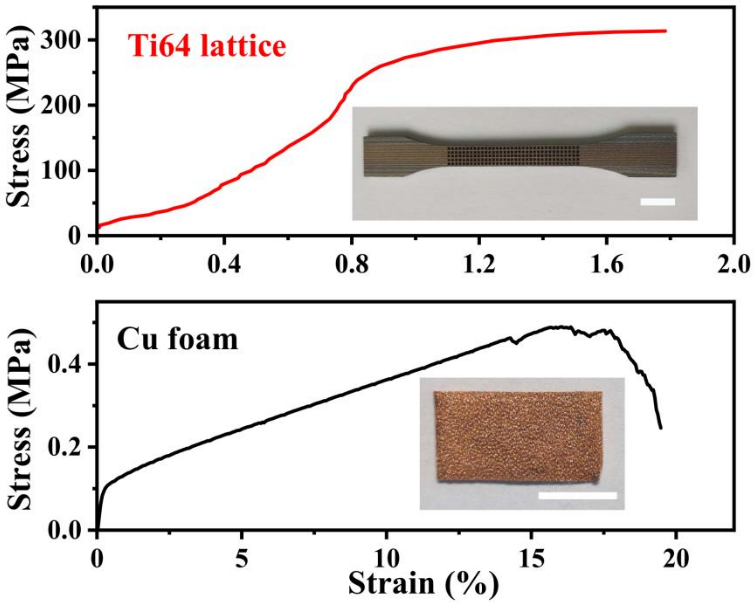

Figure S7. Stress-strain curves of the Ti64 lattice and Cu foam. The inset figures are the corresponding SLM-printed Ti64 lattice and commercial Cu foam. Scale bars are 10 mm.

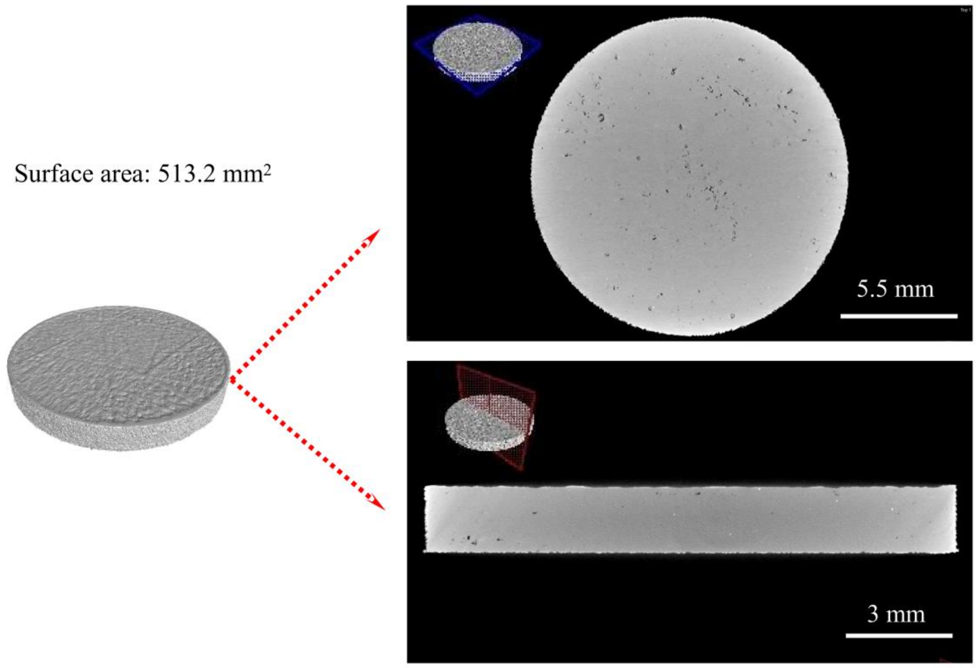

Figure S8. The micro-XCT reconstructed images of the Ti64 bulk including the top view and side view.

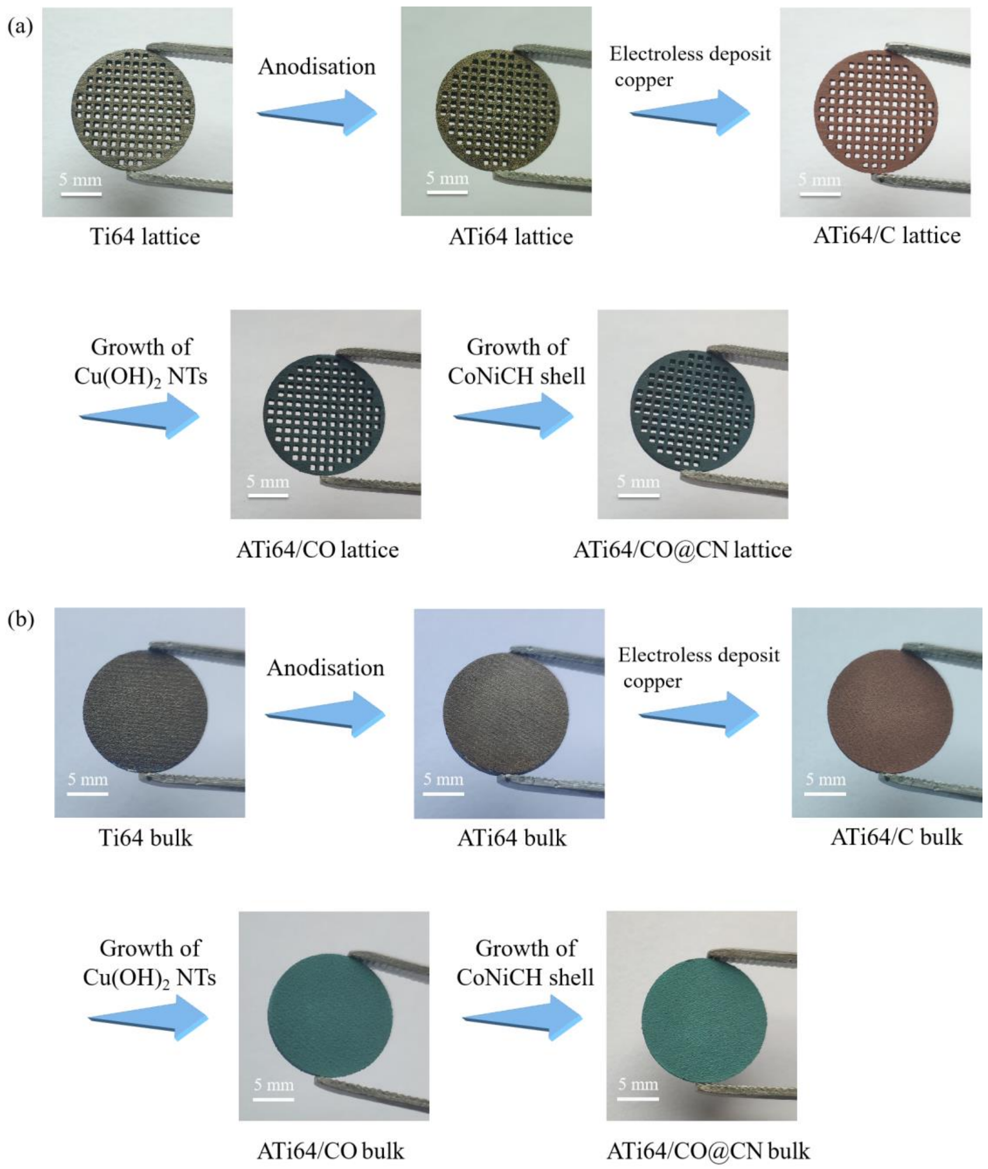

Figure S9. Digital photographs of the Ti64, ATi64, ATi64/C, ATi64/CO, and ATi64/CO@CN a) lattice and b) bulk, respectively. The Ti64 lattice (bulk) displayed an initial color of silver-gray. After the anodization, the color of the substrate was changed from silver-gray to olive-brown. The color of uniform reddish-brown was observed in the ATi64/C lattice (bulk), which suggested the successful electroless deposition of Cu. After the growth of $Cu(OH)_2$ NTs, the ATi64/CO lattice (bulk) exhibited a color of dark-cyan, and a negligible color change was found in the ATi64/CO@CN lattice (bulk).

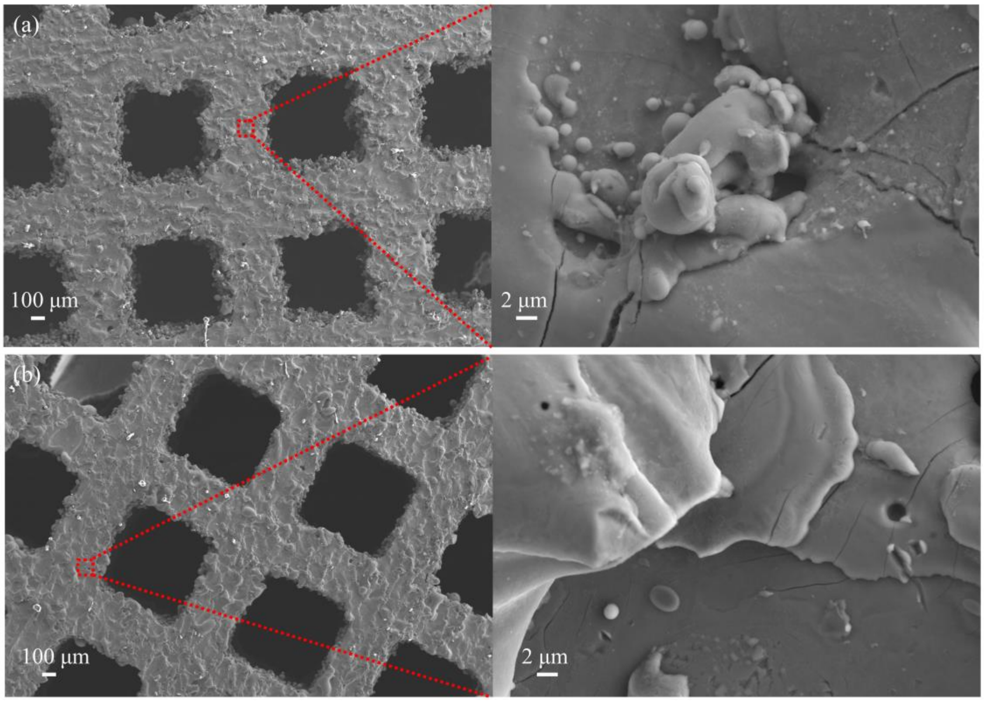

Figure S10. SEM images of the a) Ti64 lattice and b) ATi64 lattice.

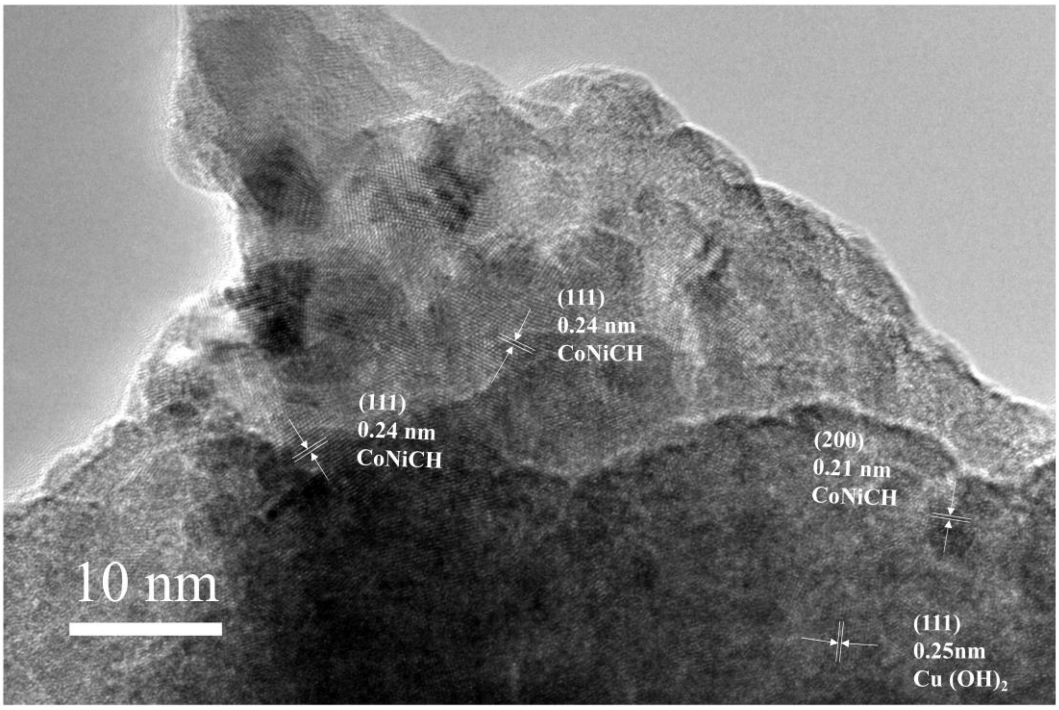

Figure S11. High-resolution TEM image of the interface structure between the $Cu(OH)_2$ NTs and CoNiCH.

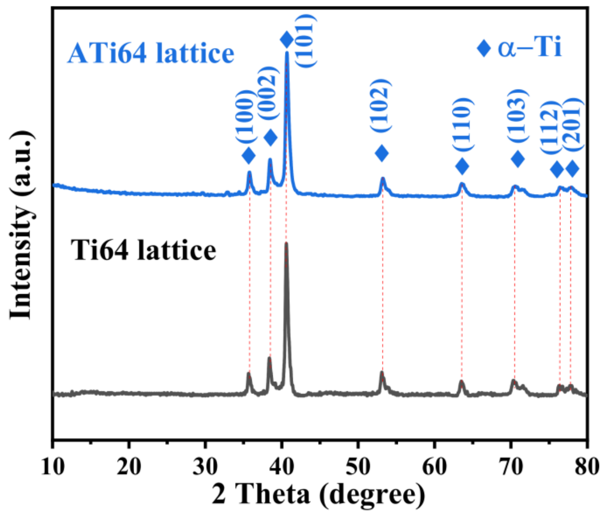

Figure S12. XRD patterns of the Ti64 and ATi64 lattice.

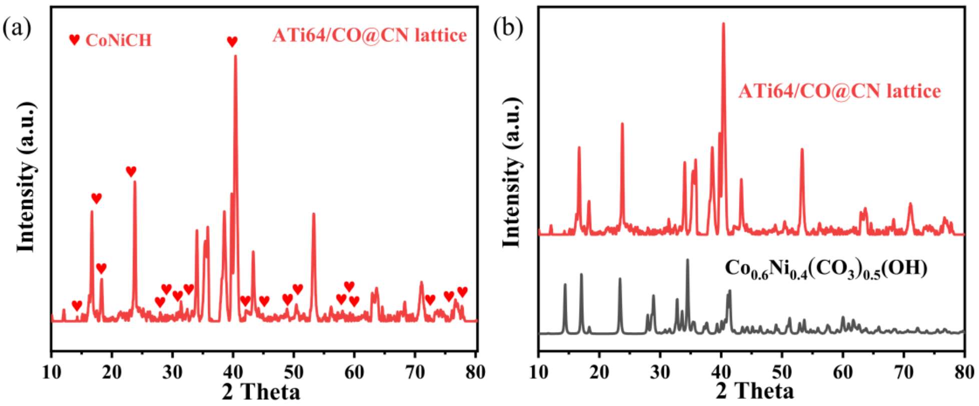

Figure S13. a) Magnified XRD pattern of the ATi64/CO@CN lattice. b) Comparison of diffraction peaks between the calculated $Co_{0.6}Ni_{0.4}(CO_3)_{0.5}(OH)$ and ATi64/CO@CN lattice.

Table S2. The element concentration of Cu, Co, and Ni in the ATi64/CO@CN lattice determined by ICP measurement. The molar ratio of Co and Ni was 1.5: 1, and the molar ratio of Co and Cu was 1.83.

Samples	Co (mg/L)	Ni (mg/L)	Cu (mg/L)
**$Cu(OH)_2$@CoNiCH**	14.5	9.82	7.91

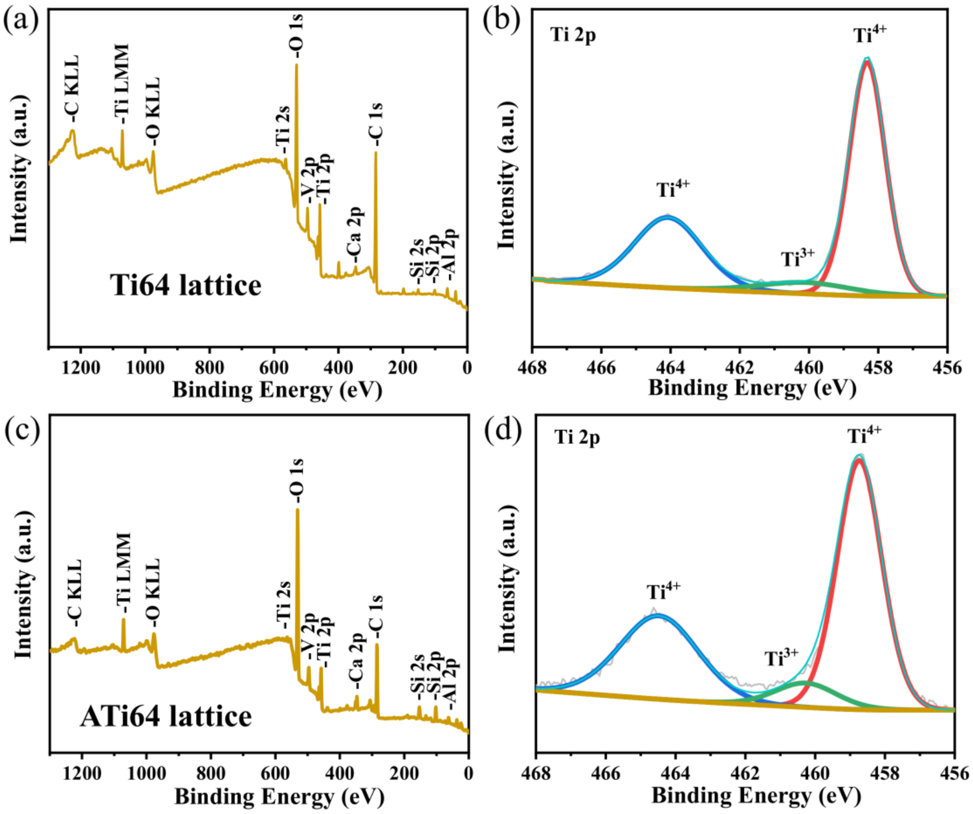

Figure S14. XPS patterns and Ti 2p spectra of the a, b) Ti64 lattice and c, d) ATi64 lattice. Both the Ti64 and ATi64 lattice exhibited the elements of Ti, Al, V, Ca, Si, C, and O, and the Ti 2p spectrum was divided into Ti^{4+} (458.7 and 464.5 eV) and Ti^{3+} (460.3 eV), corresponding to TiO_2 and Ti_2O_3, respectively.[1]

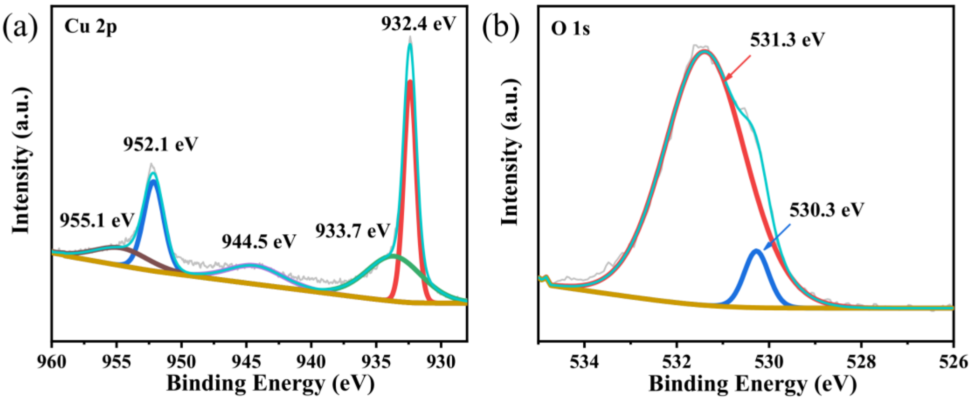

Figure S15. a) Cu 2p and b) O 1s spectrum of the ATi64/C lattice.

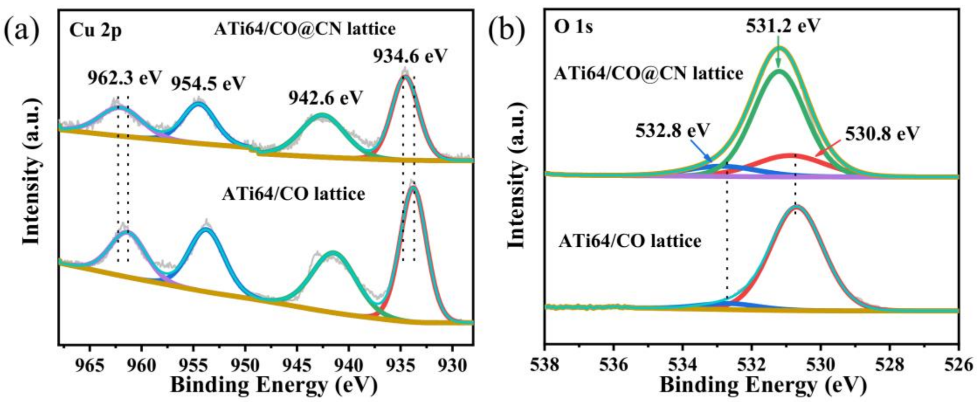

Figure S16. a) Cu 2p and b) O 1s spectrum of the ATi64/CO and ATi64/CO@CN lattice.

Table S3. The overpotentials of the as-prepared ATi64, ATi64/C, ATi64/CO, and ATi64/CO@CN lattice at various current densities.

Samples	Overpotentials (mV) at various current densities	
	**30 mA cm^{-2}**	**50 mA cm^{-2}**
**ATi64 lattice**	674	NA
**ATi64/C lattice**	571	616
**ATi64/CO lattice**	419	451
**ATi64/CO@CN lattice**	355	382

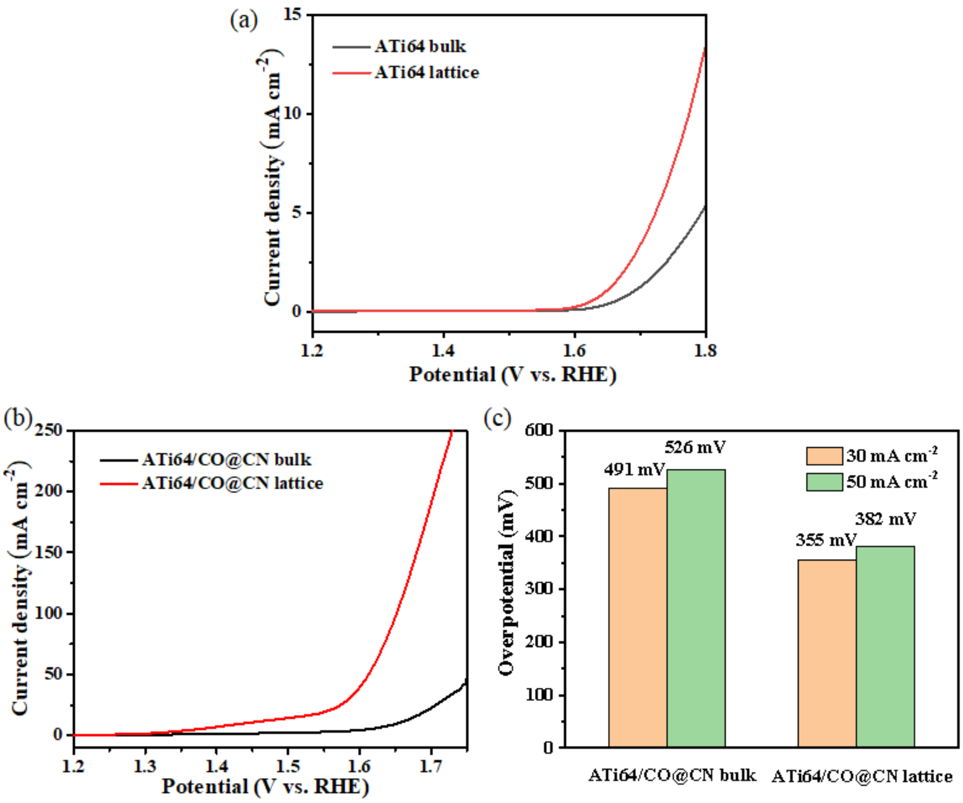

Figure S17. a) LSV curves of the ATi64 bulk and ATi64 lattice with 90% iR compensation. b) LSV curves and c) overpotentials at 30 and 50 mA cm^{-2} of the ATi64/CO@CN bulk and ATi64/CO@CN lattice with 90% iR compensation. Compared with the ATi64 bulk, the ATi64 lattice exhibited a higher electrocatalytic activity. Similarly, after the growth of $Cu(OH)_2$@CoNiCH catalysts, the ATi64/CO@CN lattice displayed overpotentials of 355 and 382 mV at 30 and 50 mA cm^{-2}, which were lower than those of the ATi64/CO@CN bulk (491 and 526 mV), respectively.

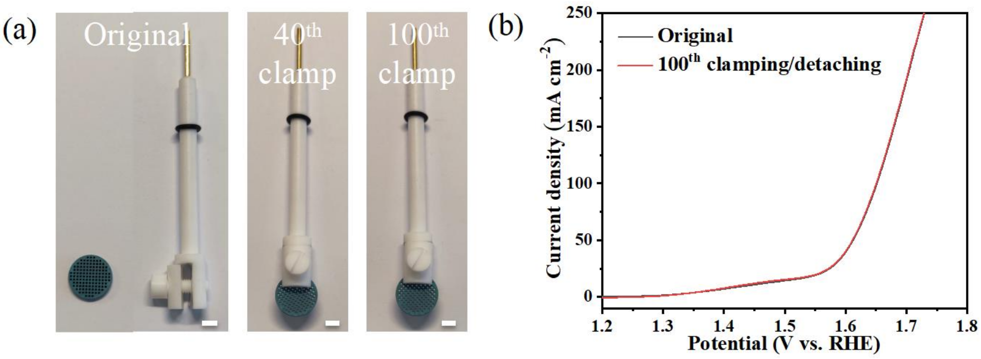

Figure S18. a) Digital photographs and b) Polarization curves of the ATi64/CO@CN lattice before and after 100th clamping/detaching, respectively. Scale bars are 5 mm.

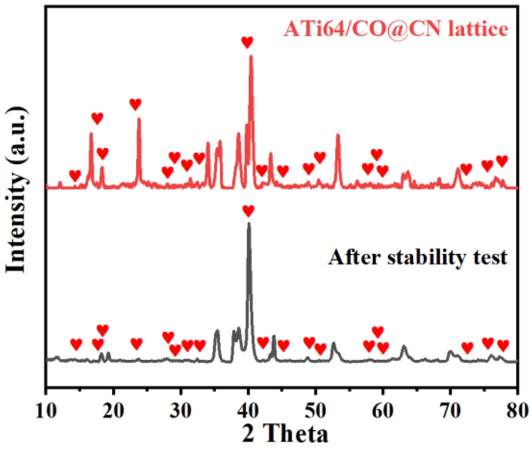

Figure S19. XRD patterns of the ATi64/CO@CN lattice before and after the stability test at 30 mA cm^{-2} for 120 h.

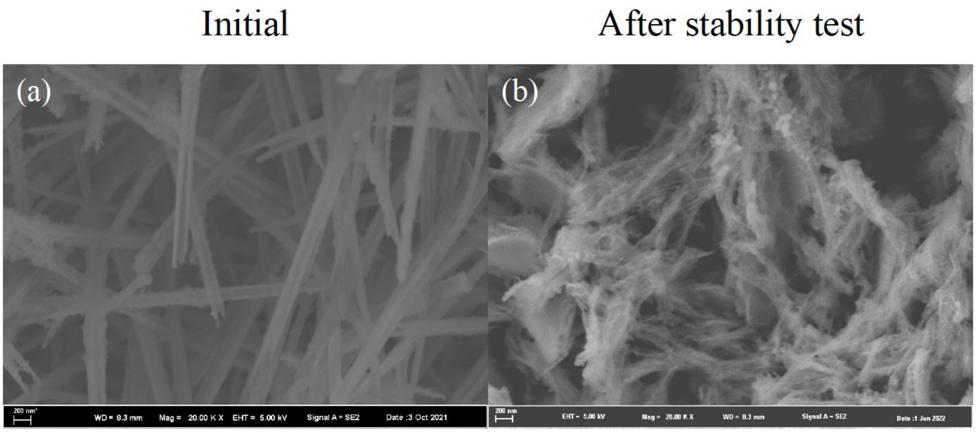

Figure S20. SEM images of the ATi64/CO@CN lattice a) before and b) after the stability test at 30 mA cm^{-2} for 120 h.

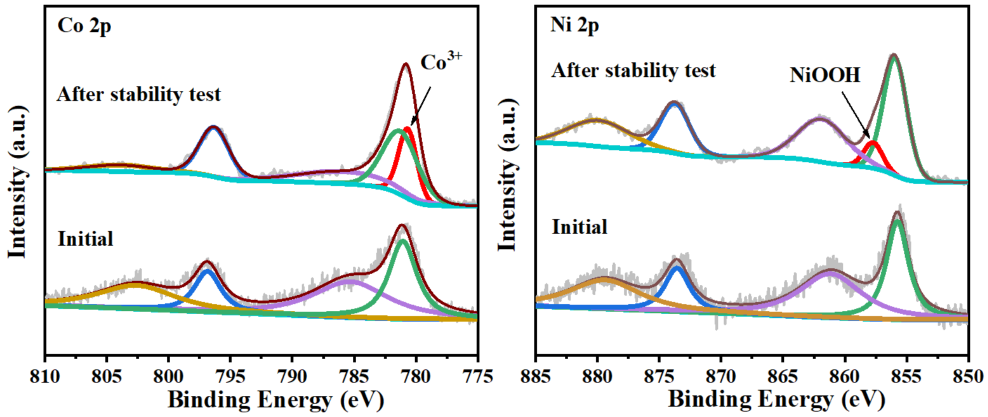

Figure S21. a) Co 2p and b) Ni 2p spectrum of the ATi64/CO@CN lattice before and after the stability test at 30 mA cm^{-2} for 120 h.

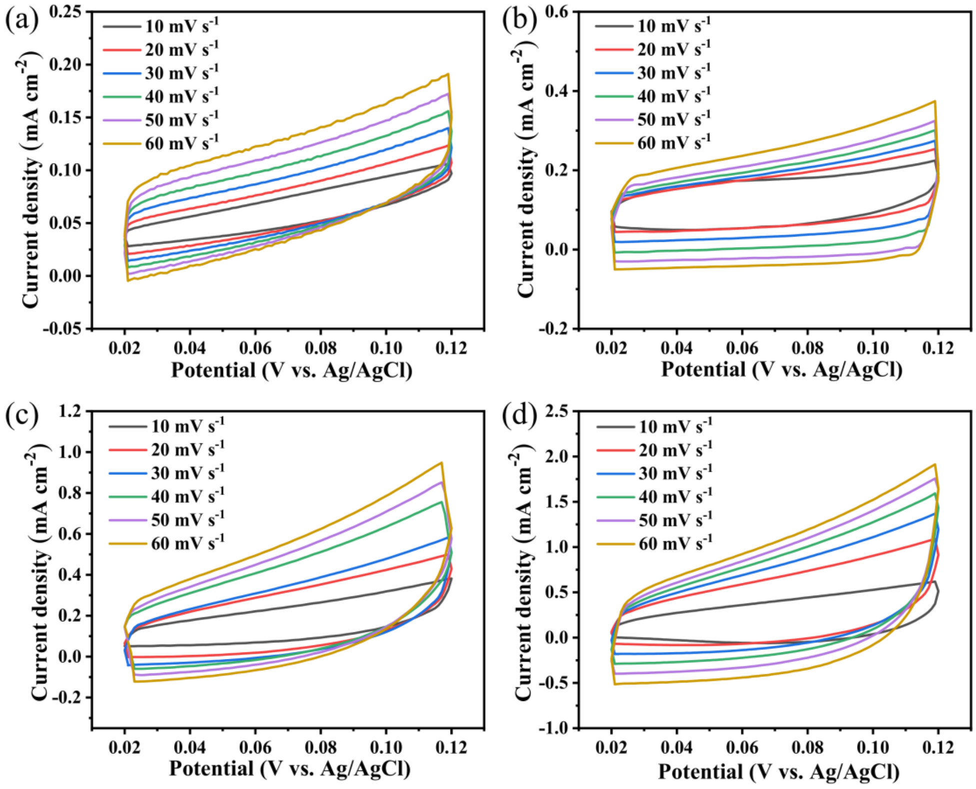

Figure S22. CV curves of the a) ATi64, b) ATi64/C, (c) ATi64/CO, and d) ATi64/CO@CN lattice at various current densities.

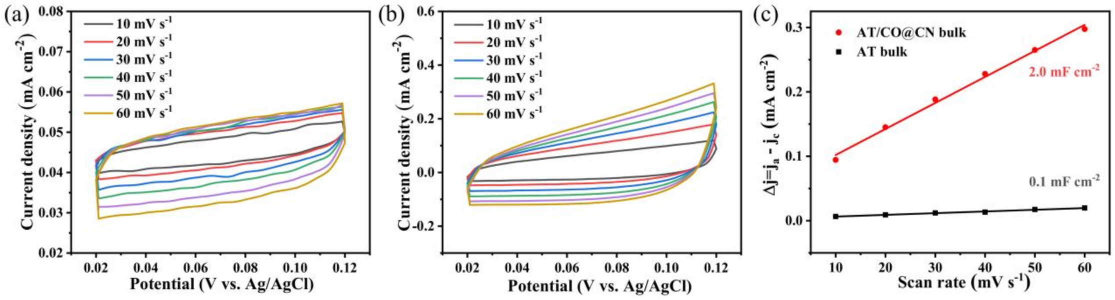

Figure S23. CV curves of the a) ATi64 and b) ATi64/CO@CN bulk at various current densities. c) Current density differences plotted as a function of the scan rates of the ATi64 and ATi64/CO@CN bulk.

Table S4. The simulated solution resistance and charge transfer resistance of the as-prepared ATi64, ATi64/C, ATi64/CO, and ATi64/CO@CN lattice.

Samples	Solution resistance (Rs/ohm)	Charge transfer resistance (Rct/ohm)
**ATi64 lattice**	5.6	453.7
**ATi64/C lattice**	2.3	65.9
**ATi64/CO lattice**	2.5	36.3
**ATi64/CO@CN lattice**	2.9	15.8

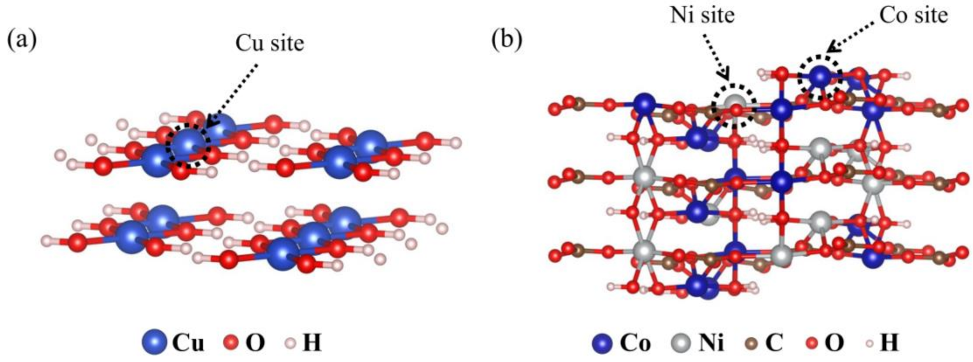

Figure S24. Atomic configurations of the a) $Cu(OH)_2$ and b) CoNiCH.

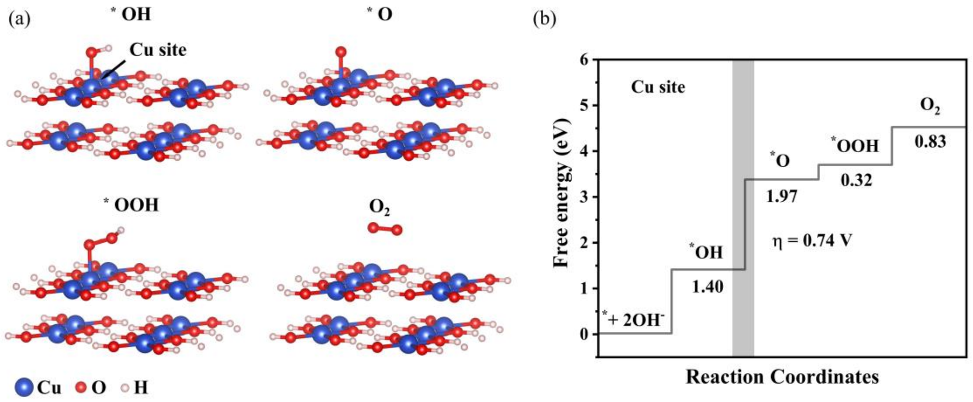

Figure S25. a) Structures of the *OH, *O, *OOH, and O_2 intermediates at the Cu site of $Cu(OH)_2$. b) Gibbs free energy graphs of OER for the $Cu(OH)_2$ with Cu site.

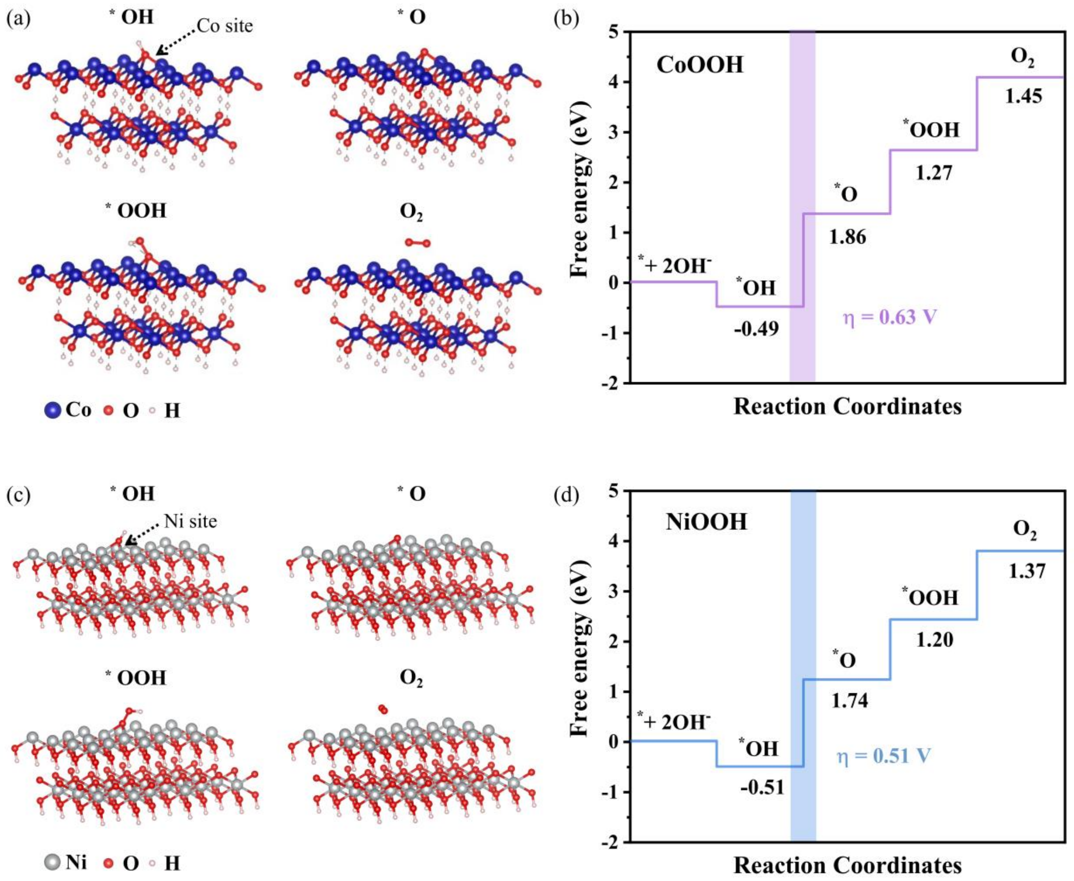

Figure S26. Structures of the *OH, *O, *OOH, and O_2 intermediates at the a) Co site of the CoOOH and c) Ni site of the NiOOH. Gibbs free energy graphs of OER of the b) CoOOH with Co site and d) NiOOH with Ni site.

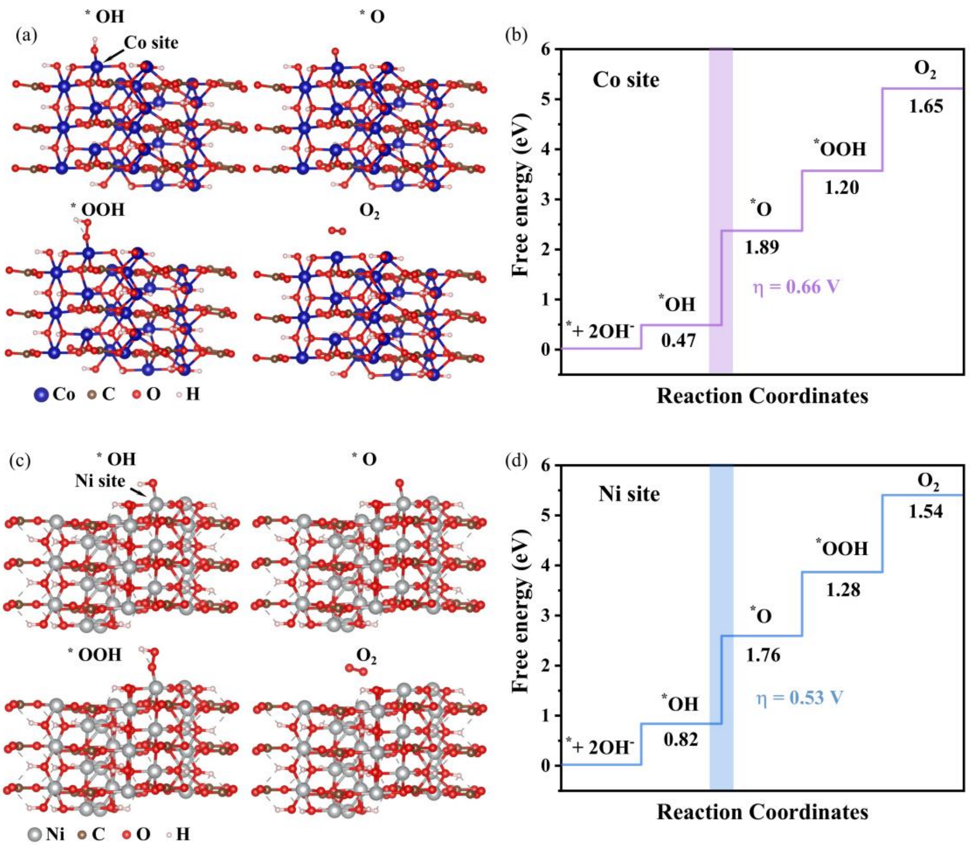

Figure S27. Structures of the *OH, *O, *OOH, and O_2 intermediates at the a) Co site of the CoCH and c) Ni site of the NiCH. Gibbs free energy graphs of OER for the b) CoCH with Co site and d) NiCH with Ni site.

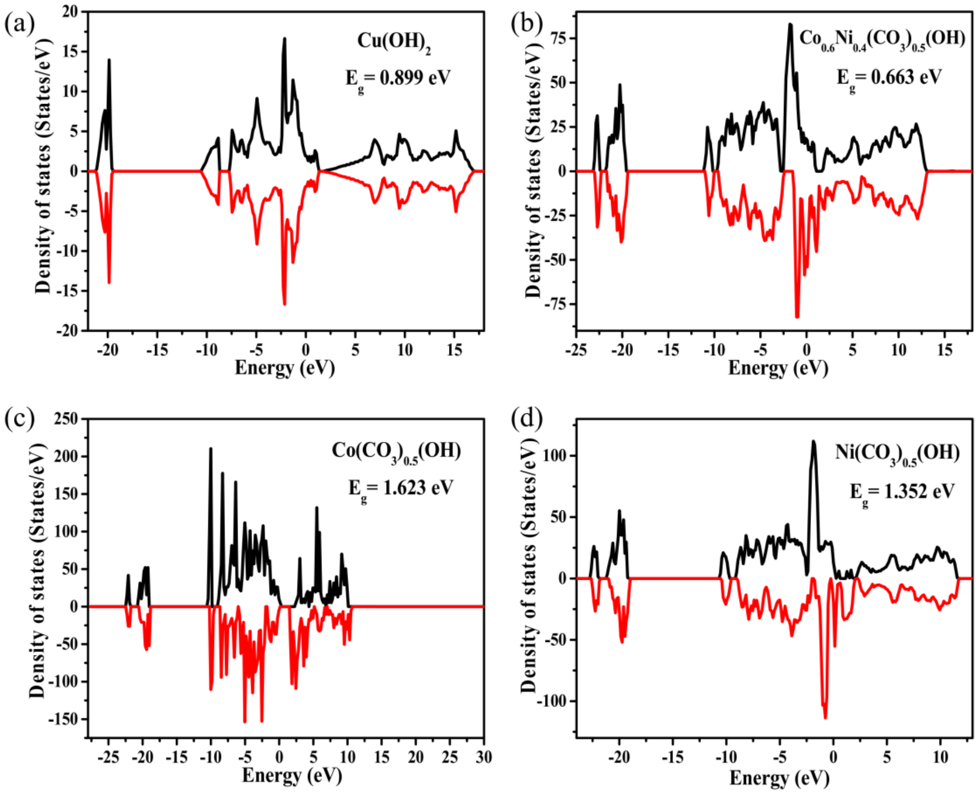

Figure S28. DOS and E_g of the a) $Cu(OH)_2$, b) CoNiCH, c) CoCH, and d) NiCH.

Reference

[1] B. Bharti, S. Kumar, H.-N. Lee, R. Kumar, *Sci. Rep.* **2016**, *6*, 1.
